# Fatty Acid Synthase Is the Key Regulator of Fatty Acid Metabolism and Is Related to Immunotherapy in Bladder Cancer

**DOI:** 10.3389/fimmu.2022.836939

**Published:** 2022-03-22

**Authors:** Qiao Xiong, Dechao Feng, Ziwei Wang, Yidie Ying, Chuanliang Xu, Qiang Wei, Shuxiong Zeng, Lu Yang

**Affiliations:** ^1^ Department of Urology, Institute of Urology, West China Hospital of Sichuan University, Chengdu, China; ^2^ Department of Urology, Changhai Hospital, Naval Medical University, Shanghai, China

**Keywords:** bladder cancer, fatty acid metabolism, FASN (fatty acid synthase), tumor immune microenvironment, immunotherapy, ceRNA network

## Abstract

Fatty acid metabolism (FAM) genes are potentially useful for predicting prognosis and immunotherapy response in bladder cancer (BC). To examine this, we constructed a prognostic model and identified key FAM genes in BC. Using transcriptional expression profiles and clinical data of BC patients from public datasets and Changhai (CH) hospital, we built and validated a risk-score model based on 13 prognostic FAM genes. Differential gene expression identified fatty acid synthase (*FASN*) as central to fatty acid metabolism in BC. FASN was differentially expressed between normal and tumor tissue, and was related to survival. In the CH dataset, FASN independently predicted muscle-invasive BC. FASN differential expression was significantly related to immune-cell infiltration and patients with low FASN expression responded better to immune checkpoint inhibitor (ICI) treatment. SREBF1 was predicted as the most significant transcription factor for *FASN*. Competing endogenous RNA network analysis suggested that lncRNA AC107027.3 may upregulate FASN by competitively binding miR-27A-3p, thereby regulating the immunotherapy response in BC. Dasatinib and temsirolimus are potential FASN-targeting drugs. Our model efficiently predicted prognosis in BC. FASN is central to fatty acid metabolism, and a potential indicator and regulator of ICI treatment.

## Introduction

Bladder cancer (BC) is among the top 10 most common cancers globally, with an estimated 573 000 new diagnoses and 212 000 deaths in 2020 (1). It has two main subtypes, non-muscle-invasive bladder cancer and muscle-invasive bladder cancer, requiring different diagnostic and treatment strategies. In recent decades, many potential biomarkers for BC diagnosis, prognosis, and therapy have been identified *via* advances in bioinformatics and sequencing (2). However, most of these have not been effective, and clinical strategies still depend mainly on pathology and imaging results (3). New approaches to identify new biomarkers for BC prognosis and therapy are urgently required.

Glucose, lipid, and protein metabolism regulates many important biological processes in cell proliferation and differentiation. Metabolic disorders can promote the tumor occurrence and progression by dysregulating the energy supply, molecular synthesis, and the microenvironment (4). Metabolomic analyses have revealed novel biomarkers related to diagnosis, prognosis and progression in many cancers, and novel antitumor strategies based on metabolism regulation have attracted attention (5). Lipids, one of the three major molecule types studied in metabolomics, are crucial in signal transduction and cellular membrane synthesis (6). The study of lipid metabolism in cancer has gone beyond classical cellular bioenergetics, opening new doors in tumor research (7). Fatty acids, the main intermediate products of lipid metabolism, participate in metabolic diseases as well as cancer genesis and development (8). For instance, reprogramming of fatty acid metabolism (FAM) by functional molecules promoted metastasis in gastric cancer (9), which is central to lipid metabolism. FAM has been targeted in chemotherapy, radiotherapy, and immunotherapy (10–12).

The energy-supply function of glucose, and the molecular functions of proteins, directly affect tumor-cell biological processes; this has increased the focus on glucose and amino acid metabolism. Glucose, proteins, and fatty acids influence each other *via* the tricarboxylic acid cycle, hence their metabolism in tumors is highly integrated. In terms of their metabolism, fatty acids, glucose, and amino acids are equally important (13). Lipid metabolism influences tumor growth and therapeutic response primarily by regulating the TME (14). Fatty acid metabolism can be catabolic or anabolic. Catabolism involves beta-oxidation, while anabolism involves biosynthesis, elongation, desaturation, and peroxidation (15). Enhancing fatty acid catabolism in multiple immune cells can re-establish antitumor function and improve the efficacy of immunotherapy (16).

Although FAM is significantly associated with BC tumor grade and stage (17, 18), prior studies have not addressed the prognostic value of FAM-related gene sets in BC. We therefore constructed a prognostic signature based on FAM genes in BC, and identified novel biomarkers for BC based on clinical features and treatment response.

## Materials and Methods

### Data Acquisition

Transcriptional expression and corresponding clinical data were obtained from The Cancer Genome Atlas (TCGA) and the Gene Expression Omnibus (GEO) databases, and were normalized and processed using the TCGAbiolinks package. FAM gene sets were collected from the Gene Set Enrichment Analysis (GSEA) website. The Changhai Hospital (CH) cohort (155 samples) was used to verify the protein expression and prognostic value of the key genes. Differential expression of the key gene was validated *via* RNA-seq of 10 paired normal and cancer tissues, and 5 tumor basal tissues, from the CH cohort. The sequencing results have been used in previous studies (19).

### Prognostic FAM Gene Identification and Subgroup Analysis

We used Venn analysis of the KEGG, HALLMARK, and REACTOME gene sets in GSEA to obtain the FAM gene set. The ggplot2 and survival R packages were used to generate heatmap plots and evaluate prognostic FAM genes. A prognostic gene coexpression network was constructed using the igraph package. A protein–protein interaction (PPI) network was generated, and hub genes identified, using STRING and Cytoscape 3.8.0.

We used the ConsensusClusterPlus package to divide the BC patients into two subgroups *via* consensus clustering, according to the 68 prognostic FAM genes. We analyzed survival and clinicopathological relatedness, and conducted a PCA of the cluster subgroups, using R. An adjusted *P* < 0.05 was considered statistically significant.

### Construction and Validation of the FAM-Related Gene Model

From the TCGA database, we randomly divided Urothelial Bladder Carcinoma (BLCA) sample patients into training and testing cohorts, then circularly performed least absolute shrinkage and selection operator (LASSO) regression and stepwise multivariate Cox regression, to develop an efficient signature. The samples were classified into high- or low-risk groups, using the median risk score. To assess the efficiency of the risk-score model, we analyzed the area under the ROC curve, performed Kaplan–Meier (KM) analysis, and generated risk plots using the training, testing, and combined cohorts. We used univariate and multivariate Cox regression analyses, with risk, cluster, age, sex, stage, and grade as factors, and constructed a nomogram. To evaluate the net benefit of the nomogram, we used the C-index, ROC curve, calibration curve, and decision curve analysis.

After validating the risk-score model, we applied copy number variation (CNV) and tumor burden mutation analysis. Standard CNV data were obtained from the NCI Center for Cancer Genomics (GDC)-TCGA cohort, and were analyzed using strawberry Perl and the RCircos package. The correlation between CNV and immune infiltration was analyzed *via* TIMER. The BC patients’ somatic mutation data were downloaded from the TCGA-BLCA database and analyzed using the maftools package.

### Identification and Validation of the Key Gene

To screen key FAM genes, we used a Venn plot to analyse PPI hub genes, our model-identified genes, and TCGA DEGs. The mutation atlas of the key gene was downloaded from the cBioPortal database (https://www.cbioportal.org/). The three dimensional structure of the protein translated by the key gene, and the location atlas, were obtained from the Protein Data Bank (https://www.rcsb.org/structure/6NNA). Immunohistochemistry and immunofluorescence data were acquired from the Human Protein Atlas database (https://www.proteinatlas.org).

We first confirmed the differential expression, pathological correlation, and prognostic value of the key genes using six public datasets (TCGA, GSE13507, GSE3167, GSE40355, GSE32548, and GSE32894) (20–24). External validation was performed using the CH cohort. Ten paired samples of RNA-seq and IHC data were used to verify differential expression, and another 155 IHC-stained cancer samples were used to evaluate the clinical prognostic value of the key gene. IHC staining chips for the key gene were visualized using Image-Pro Plus (IPP) software, and assessed by a professional pathologist.

### GSEA and Gene Set Variation Analysis (GSVA)

We divided TCGA BC patients into low- and high key-gene expression groups, and conducted Kyoto Encyclopedia of Genes and Genomes (KEGG) and Gene Ontology (GO) analyses. GSVA was applied to estimate pathway scores for the low and high expression groups (25). We used the R packages GSEABase, GSVA, and limma, and considered *P* < 0.05 as statistically significant. GSEA was used to identify significantly upregulated and downregulated pathways (26), using software downloaded from the Broad Institute, as well as the R packages org.Hs.eg.db, GOplot, digest, and enrichplot. Statistical significance was set at a |normalized enrichment score| > 1, nominal *P* < 0.05, and FDR q < 0.25.

### Immune Function of the Key Gene

On the basis of GSVA and GSEA, we selected immune infiltration as the process for further analysis, and downloaded original immune infiltration data from TIMER2.0. We then applied seven algorithms (TIMER, CIBERSORT, CIBERSORT-ABS, QUANTISEQ, MCPCOUNTER, XCELL, and EPIC) in the limma package, using the Wilcoxon test (*P* < 0.05). We further compared the high- and low-expression groups in terms of immune cell infiltration, immune function, immune microenvironment, and immune checkpoints. We downloaded information on the therapeutic responses of PD-1 and CTLA-4, crucial targets of immune checkpoint inhibitor (ICI) therapy, from The Cancer Immunome Atlas (TCIA) database (https://tcia.at/home/).

### Molecular Regulation Mechanism of the Key Gene

To determine the pathway potentially regulating the transcription and translation of the key gene in BC, we analyzed transcription factors (TFs) and competing endogenous RNA (ceRNA) networks. Sequence information about the key gene was obtained from the Gene Module in the National Center for Biotechnology Information (NCBI). The region from 2000 bp before to 100 bp after the transcription start point served as the potential binding region. We predicted the potential TFs using the Genome Database of the University of California (Santa Cruz), and obtained the TF binding sequence logo and the transcription factor flexible model nucleotide correlation logo from the JASPAR database (27, 28). Next, we selected microRNAs (miRNAs) and long noncoding RNAs (lncRNAs) that may regulate the key gene from the STARBASE database, and constructed a ceRNA network using the reshape2 package and Cytoscape (29). The expression of the selected TFs, miRNAs, and lncRNAs, and their prognostic relevance, were validated using the TCGA or CH cohorts.

### Drugs Targeting the Key Gene

Based on the regulatory pathway, we screened drugs targeting the key gene. Drug sensitivity information was obtained from the CellMiner database and Gene Set Cancer Analysis (GSCA) database, which collects drug-sensitivity data from the Cancer Therapeutics Response Portal (CTRP) and the Genomics of Drug Sensitivity in Cancer (GDSC) database (30, 31). We used Venn analysis to select specific drugs targeting the key gene, and applied the pRRophetic package to compare the high- and low-expression groups in terms of their IC_50_ values. We obtained the chemical structure and clinical study information for the predicted drugs from the canSAR Black database.

### Statistical Analysis

R software v. 4.1.1 (https://www.r-project.org) and GraphPad Prism 7.0 (https://www.graphpad.com/) were used for statistical analysis. *P* < 0.05 was defined as statistically significant. We used unpaired Student’s *t*-tests and Wilcoxon tests to analyse normally and nonnormally distributed variables, respectively. The paired Student’s *t*-test was used to analyse key-gene differential expression in paired samples. Univariate, multivariate, and Lasso-penalized regression analyses were used to identify the important genes and characterize the prognostic signature.

## Results

### Selection of a Potential Prognostic Signature Based on FAM Genes in BC

**Scheme 1:**
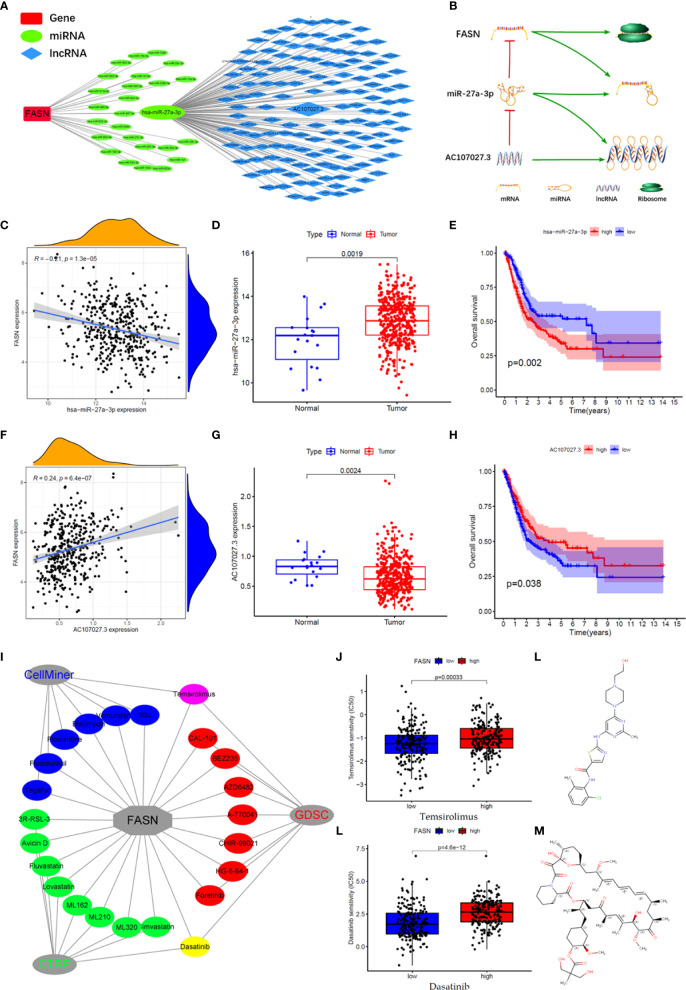
Flowchart of the analysis process.

**Figure 1:**
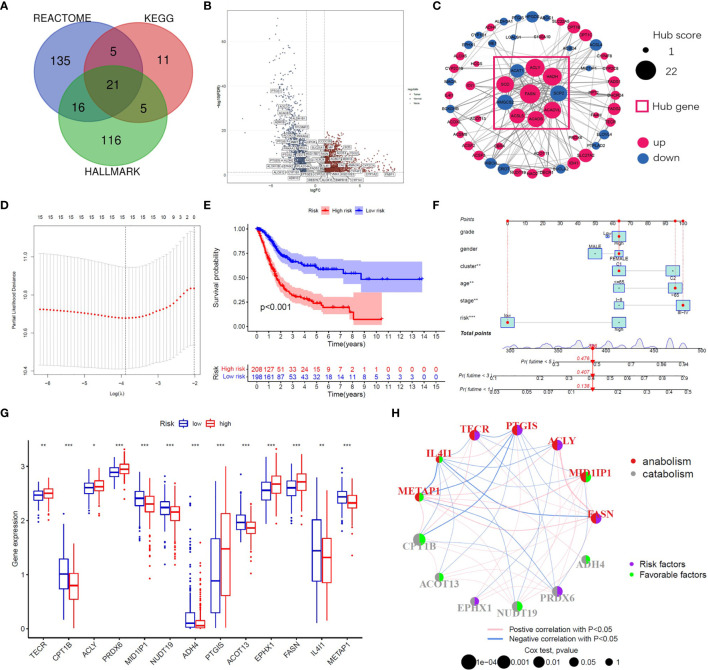
Identification of prognostic FAM genes and risk score model. **(A)** Venn diagram of 309 FAM genes from KEGG, HALLMARK and REACTOME. **(B)** Volcano plot of 52 fatty acid metabolism-related DEGs (p<0.05). **(C)** PPI network and hub genes of 68 prognostic FAM genes. **(D)** Cross-validation for tuning the parameter selection in the LASSO regression. **(E)** Kaplan–Meier survival curve of the patients between the high- and low-risk groups. **(F)** Nomogram containing risk, cluster and clinicopathological features. **(G)** Differential expression of the 13 model genes between different risk groups. **(H)** Risk and functional groups of the 13 model genes. (*p < 0.05, **p < 0.01, ***p < 0.001).

A flow chart of this study including two main parts was provided in **Scheme 1**. In total, 309 FAM genes were identified *via* Venn analysis of three functional gene sets, and 52 differentially expressed FAM genes were identified (**Figures 1A, B**). PPI analysis revealed the interactions among 68 prognostic FAM genes, *via* univariate Cox regression. The Cyto-Hubba algorithm, which is based on the degree method, revealed the top 10 hub genes (FASN, HADH, ACLY, ACADVL, SCD, ACADS, SCP2, HMGCS2, ACSL5, and ACAT1), of which FASN was the most noteworthy (**Figure 1C**). The FAM gene analysis process and co-expression network is shown in **Figures S1A–D**. Primary function analysis confirmed that the selected genes were enriched mainly in FAM pathways (**Figures S1E–H**). Consensus clustering separated the samples into two distinct subgroups (**Figure S2**).

To construct the FAM-related signature, we selected 13 genes (CPT1B, FASN, MID1IP1, ACOT13, ACLY, NUDT19, TECR, PTGIS, ADH4, PRDX6, IL4I1, EPHX1, and METAP1) using LASSO regression and multivariate Cox regression (**Figures 1D, E** and **Table S1**). The nomogram suggested that the signature was an independent prognostic factor in BC patients (**Figure 1F**). The process for building and validating the risk-score model is provided in the supplementary materials (**Figures S3**, **S4**). For further analysis, we assigned these candidate genes to catabolism and anabolism groups based on GeneCard annotation, and performed function and survival analysis (**Figures 1G, H**, **S5, S6**).

### Identification and Validation of FASN in FAM

**Figure 2:**
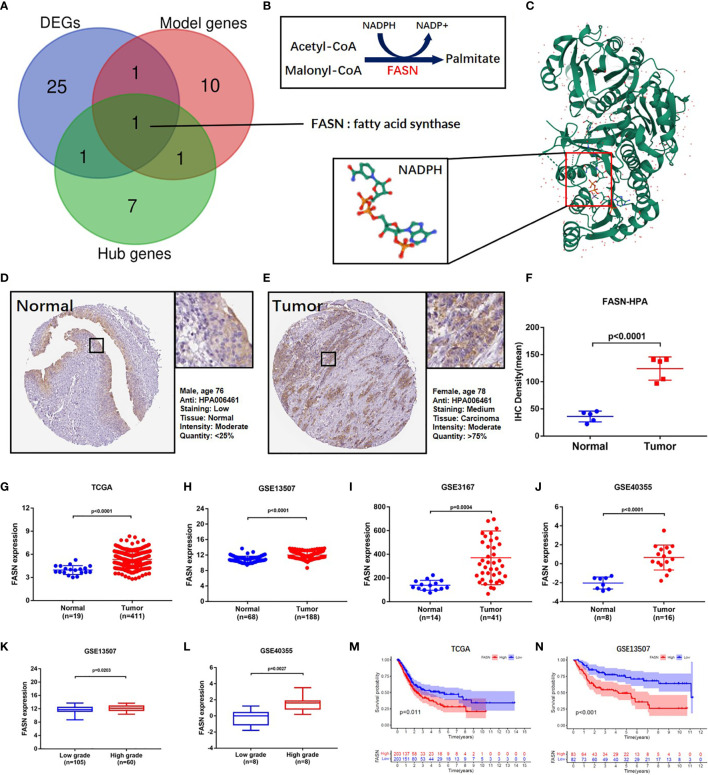
Identification and validation of FASN. **(A)** Venn diagram of the top 10 PPI hub genes, 52 differentially expressed genes and 13 model genes. **(B)** Mechanism diagram of the role of FASN in fatty acid metabolism. **(C)** 3D structure of the protein translated by FASN and NADPH ligand from the PDB database. **(D–F)** IHC staining of FASN in tumor and normal tissues of BC patients from the HPA database. **(G–J)** Differential expression of FASN between the tumor and normal groups in TAGA, GSE13507, GSE3167 and GSE40355. **(K, L)** Differential expression of FASN between low- and high-grade patients in GSE13507 and GSE40355. **(M, N)** Overall survival probability between the low- and high-FASN groups in TCGA and GSE13507 (patients grouped by median value).

Venn analysis of the 13 model-identified genes, 10 PPI hub genes, and 52 DEGs, identified fatty acid synthase (FASN) as the key FAM gene in BC patients (**Figure 2A**). Its primary function and 3D structure were obtained from the GeneCard and Protein Data Bank databases (**Figures 2B, C**). IHC staining revealed that FASN expression was higher in tumor tissues than in normal tissues, and was mainly located in the cytoplasm (**Figures 2D–F**). FASN mutation data and immunofluorescence images are presented in **Figures S7A, B**.

Based on analysis of the TAGA, GSE13507, GSE3167, and GSE40355 datasets, FASN was differentially expressed between cancer and normal tissue (**Figures 2G–J**). Clinicopathology grouping analysis, using the GSE3167, GSE40355, GSE32548, and GSE32894 datasets, indicated that FASN was highly expressed in patients with high tumor grades (**Figures 2K, L**, **S7C, D**). Kaplan-Meier analysis of TCGA data revealed that FASN showed prognostic significance, regardless of whether grouping was based on the median or optimal cutoff value (**Figures 2M**, **S7E**). Using the GSE13507 dataset, FASN expression was valuable in predicting both overall and disease-free survival of BC patients (**Figures 2N**, **S7F**).

### External Validation of FASN

**Figure 3:**
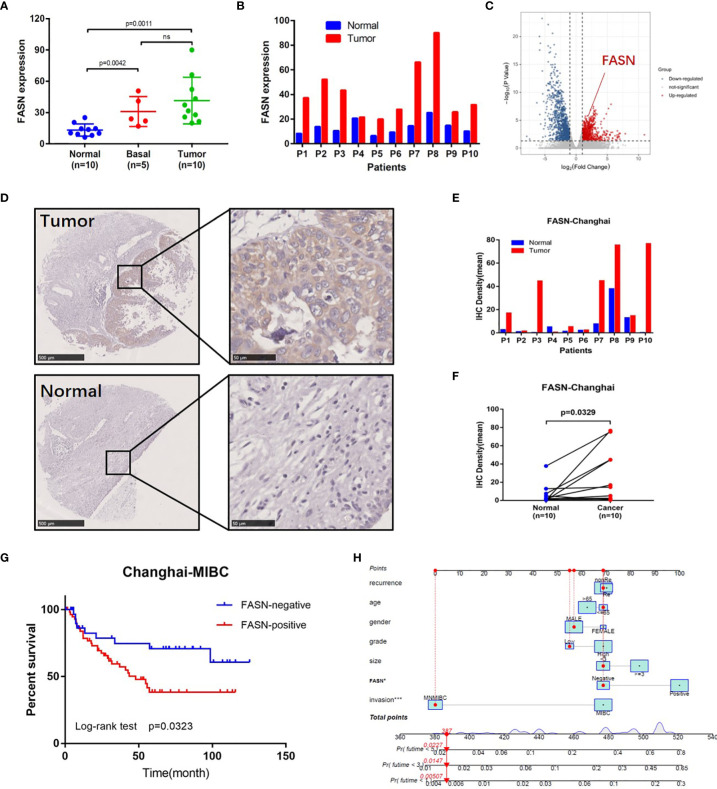
External validation of FASN. **(A)** Differential expression of FASN in normal, basal and tumor tissues. **(B)** Differential expression of FASN in 10 paired normal and tumor tissues. **(C)** Volcano plot of paired tissue RNA-seq matrix (logFC>1, p<0.05). **(D–F)** IHC staining of the 10 paired normal and tumor tissues. **(G)** Overall survival curve between the positive and negative FASN groups of MIBC patients in the CH cohort. **(H)** Nomogram with FASN and clinicopathologic features for the prediction of outcome in the CH cohort.

External validation of FASN (in the CH cohort) used 25 sequencing samples and 155 tissue samples. Based on RNA-seq data, FASN expression appeared to be lower in normal tissues than basal and tumor tissues (**Figure 3A**). Paired-sample analysis revealed higher FASN expression in tumor than normal tissues, in all 10 patients (**Figure 3B**). The volcano plot further indicates that FASN was significantly upregulated in tumor tissues (**Figure 3C**). IHC staining of the 10 paired tissue samples confirmed that FASN expression was higher in tumor tissues (**Figures 3D–F**, **S8A**).

IHC staining of FASN was conducted using 155 tissue chips. The IHC score was calculated as the product of the staining intensity (0–3) and proportion (0–100) (**Figure S8B, C**). The patients were divided into FASN-negative and -positive groups by IHC score, for further analysis (**Table S2**). Kaplan-Meier analysis of overall survival showed that FASN was prognostic in muscle-invasive bladder cancer (n = 92) but not in non-muscle-invasive bladder cancer (n = 63) (**Figures 3G**, **S8F**). For muscle-invasive bladder cancer patients, the median survival time of the FASN-positive group was 47.93 months (that of the FASN-negative group was not available). We found no correlation between FASN expression and tumor invasion and grade (**Figure S8D, E**), although multivariate Cox regression analysis revealed that FASN and invasion were both independent prognostic factors (**Table 1**). We further constructed nomograms to predict the 1-, 3- and 5-year BC survival. Adding FASN to the nomogram increased its accuracy from 0.738 to 0.762, suggesting that FASN is an important prognostic factor (**Figure 3H**).

**Table 1:** The results of univariate and multivariate COX regression analysis in BC patients in the CH cohort.

Features	Univariate analysis				Multivariate analysis			
	HR	HR.95L	HR.95H	P value	HR	HR.95L	HR.95H	P value
Age (<=65 vs >65)	1.04	1.00	1.07	0.0271	1.02	0.99	1.06	0.1718
Gender (male vs female)	0.95	0.37	2.42	0.9176	0.60	0.22	1.61	0.3083
Recurrence (re vs non-re)	1.12	0.60	2.08	0.7314	1.29	0.68	2.47	0.4380
Size (<3 vs >=3)	1.70	0.90	3.22	0.1012	1.25	0.64	2.44	0.5075
Grade (high vs low)	1.01	0.62	1.67	0.9561	0.82	0.45	1.51	0.5271
Invasion (NMIBC vs MIBC)	7.96	3.13	20.27	0.0000	7.85	2.98	20.70	0.0000
FASN (negative vs positive)	1.89	0.93	3.83	0.0775	2.55	1.19	5.49	0.0164

### GSEA and GSVA

**Figure 4:**
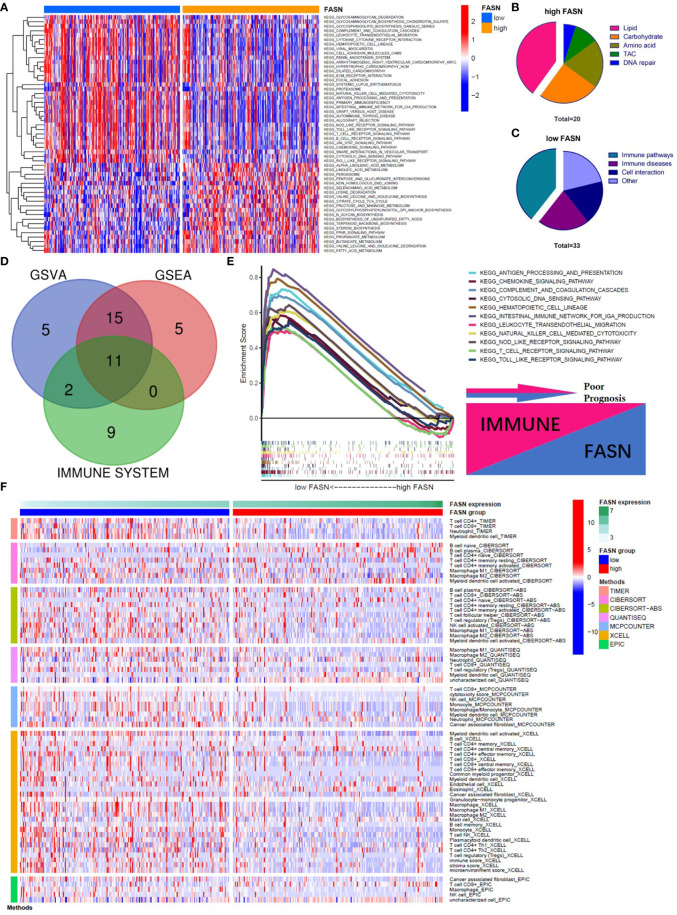
Function analysis of FASN. **(A)** Heatmap of KEGG enrichment for the low- and high-FASN groups in GSVA (p<0.05). **(B)** Types of KEGG pathways enriched in the high-FASN group. **(C)** Types of KEGG pathways enriched in the low-FASN group. **(D)** Venn plot of pathways enriched in low-FASN group. **(E)** Gene enrichment of the 11 selected immune pathways in GSEA. **(F)** Heatmap of immune infiltration between low- and high-FASN groups by seven algorithms (p<0.05).

In the KEGG analysis, GSVA and GSEA identified 53 and 39 enriched functional pathways, respectively. In the high-FASN group, most of the functional pathways were related to metabolism, whereas in the low-FASN group, they were mostly immune-related (**Figures 4A–C**). Intriguingly, 11 of the 22 immune system KEGG pathways were significantly enriched in the low-FASN group, both *via* GSEA and GSVA (**Figures 4D, E**). GO analysis yielded similar results (**Figure S9**). As both KEGG and GO analysis indicated that low FASN expression is associated with immune function, we conducted a correlation analysis between immune-cell infiltration and FASN expression. The heatmap revealed significant differences in immune-cell infiltration between the low- and high-FASN groups, for multiple databases (**Figure 4F**).

### Immune Function of FASN

The functional analysis indicated that many immune pathways were enriched in the low-FASN group. We therefore also analyzed immune cell infiltration, immune function, immune microenvironment, and immune checkpoints, to explore the role of FASN in cancer immune infiltration and responses.

**Figure 5:**
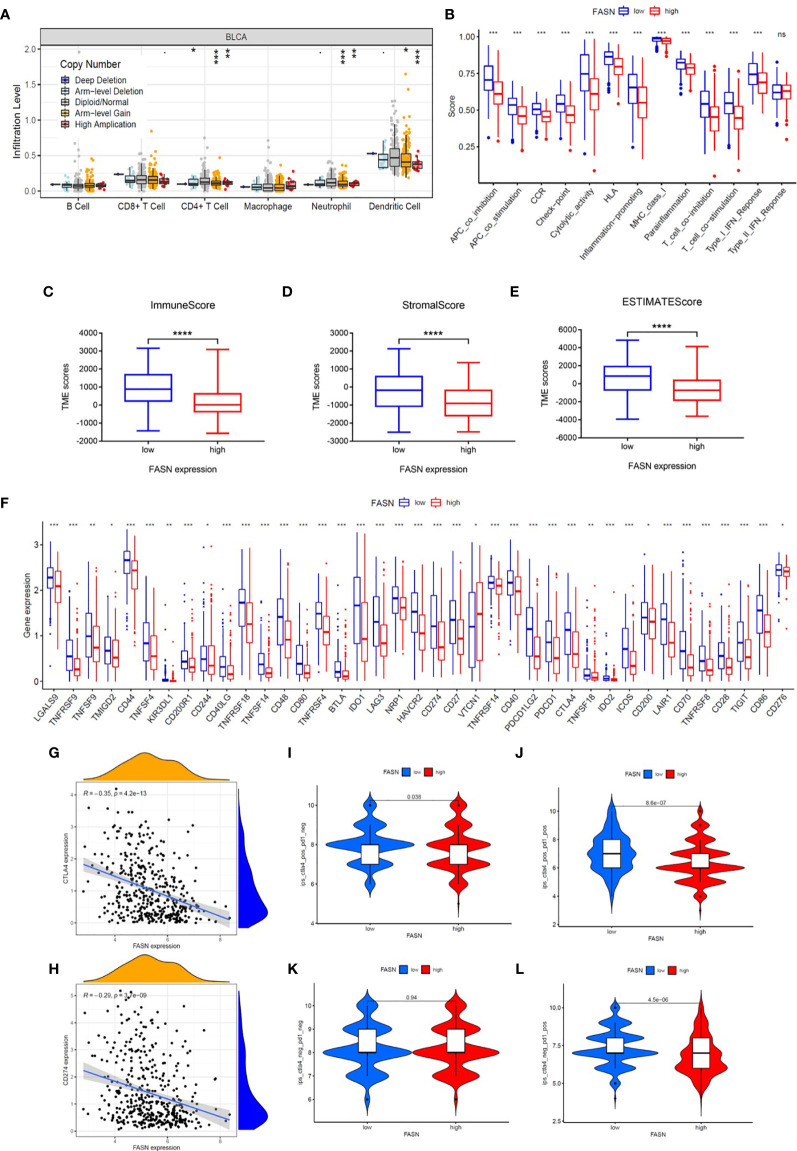
Immune function of FASN. **(A)** Effect of FASN CNV status on immune cell infiltration. **(B)** ssGSEA scores of immune function between the low- and high-FASN groups. **(C–E)** Boxplot of TME score between low- and high-FASN groups. **(F)** Differential expression of 39 immune checkpoints between low- and high-FASN groups. **(G, H)** Scatter plot of correlation between CD274 (PD-L1), CTLA4 and FASN. **(I–L)** ICI treatment response of low and high-FASN groups. (ns p > 0.05, *p < 0.05, **p < 0.01, ***p < 0.001, ****p < 0.0001).

CIBERSORT analysis revealed that five types of immune cells were differentially infiltrated between the low- and high-FASN groups and FASN CNV significantly affects the infiltration of CD4+ T cells, dendritic cells, and neutrophils (**Figures 5A**, **S10A**). Further correlation analysis of the expression of FASN and immune cell marker genes indicated that six types of immune-cell infiltrate were associated with FASN expression (**Figures S10B, C**). Single-sample GSEA scores of immune function revealed that all of the immune function indicators were significantly higher in the low-FASN group, except for the type II INF response (**Figure 5B**). The low-FASN group had higher stromal, immune, and ESTIMATE TME scores (**Figures 5C–E**). In particular, most of the immune checkpoints (39/48), including CD274 (PD-L1) and CTLA4, were differentially expressed between the high-and low-FASN groups (**Figure 5F**). Evaluation of immune checkpoint therapy, based on the TCIA database, revealed that patients with low FASN levels responded better to anti- PD-1 and CTLA4 treatments than those with high FASN levels (**Figures 5G–L**)

### Molecular Regulation of FASN

**Figure 6:**
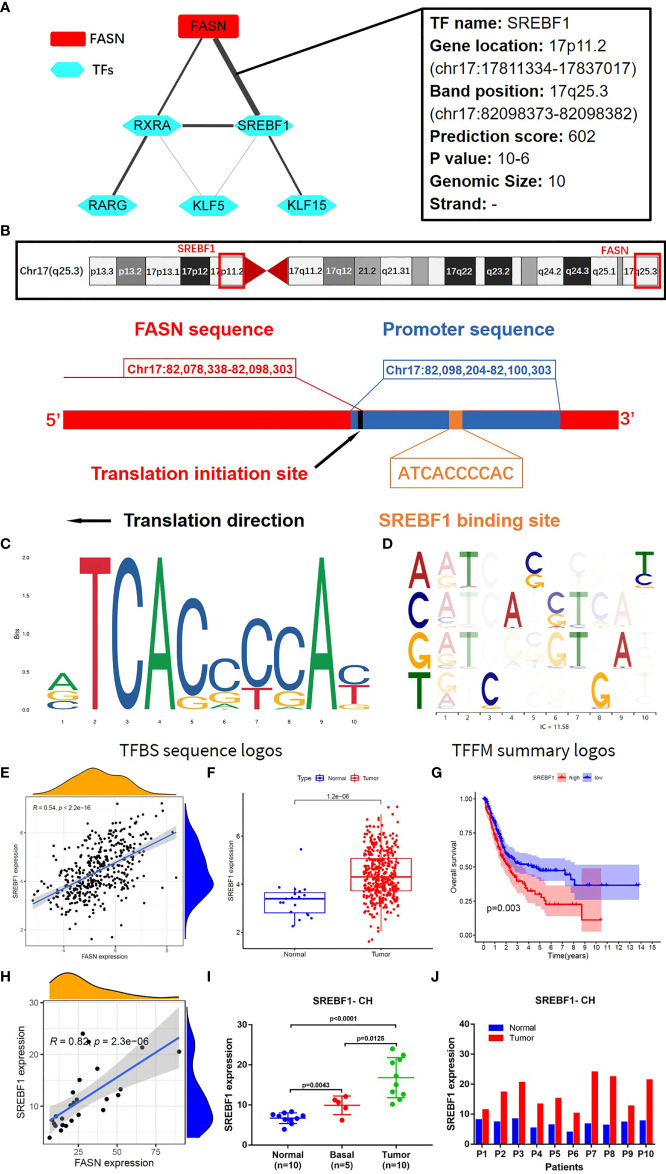
Transcription factors regulating FASN. **(A)** Correlation network of FASN and TFs. **(B)** Diagram of gene location and transcription of FASN and SREBF1. **(C)** TFBS sequence logos of SREBF1 (the ordinate represents the amount of base information, and the abscissa represents the base location). **(D)** Base correlation logos of TFBS by TFFM. **(E)** Scatter plot of association between FASN and SREBF1 in TCGA. **(F)** Boxplot of SREBF1 expression in normal and tumor patients in TCGA. **(G)** Overall survival curve of low- and high-SREBF1 groups in TCGA. **(H)** Scatter plot of association between FASN and SREBF1 in CH database. **(I)** Differential expression of FASN in normal, basal and tumor tissues. **(J)** Differential expression of FASN in 10 paired normal and tumor tissues.

TF analysis revealed a potential regulatory mechanism of FASN in the nucleus. Fifteen TFs in the JASPAR CORE collection (2022) were predicted to be associated with FASN transcription, with a minimum score > 600. Correlation analysis suggested that sterol regulatory element binding transcription factor 1 (SREBF1) was the TF most significantly related to FASN (**Figure 6A**). SREBF1 and FASN are both located on chromosome 17; the TF binding site is shown in **Figure 6B**. The binding sequence logo and nucleotide correlation logo generated by transcription factor flexible models are presented in **Figures 6C, D**. Our analysis of association, expression, and prognosis revealed that SREBF1 was highly correlated with FASN, and was also prognostic in BC patients (**Figures 6E–G**). Analysis of RNA-seq data from the CH cohort validated that SREBF1 was associated with FASN, and was differentially expressed in normal and tumor tissues (**Figures 6H–J**).

**Figure 7:**
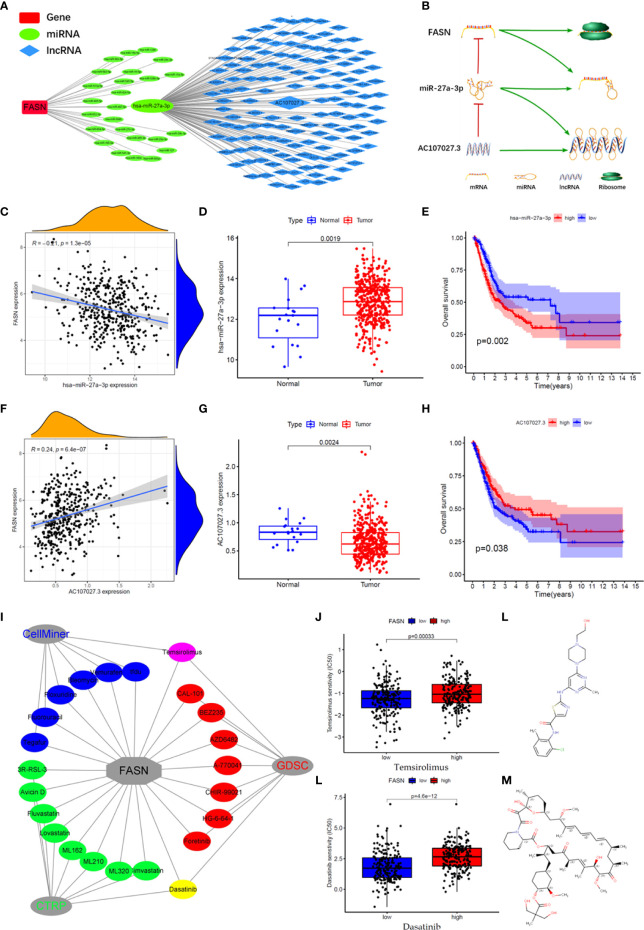
CeRNA network and drugs prediction of FASN. **(A)** mRNA-miRNA-lncRNA interaction network of FASN in BC. **(B)** AC107027.3 competitively binds miR-27a-3p to decrease its inhibition on FASN. **(C)** Scatter plot of association between FASN and miR-27a-3p in TCGA. **(D)** Boxplot of miR-27a-3p in normal and tumor patients in TCGA. **(E)** Overall survival curve of low- and high- miR-27a-3p groups in TCGA. **(F)** Scatter plot of association between FASN and AC107027.3 in TCGA. **(G)** Boxplot of AC107027.3 in normal and tumor patients in TCGA. **(H)** Overall survival curve of low- and high- AC107027.3 groups in TCGA. **(I)** Drugs targeting FASN in the CTRP, GDSC, and CellMiner databases. **(J, K)** IC50 of dasatinib and temsirolimus in low- and high-FASN groups. **(L, M)** Chemical structure of dasatinib and temsirolimus from canSARblack.

We constructed an mRNA–miRNA–lncRNA network to examine the translational regulation of FASN in the cytoplasm (**Figures 7A, B**). First, 26 miRNAs potentially targeting FASN were obtained from the STARBASE database, and miR-27a-3p was identified *via* Spearman correlation analysis (**Figure 7C**). The expression boxplot and overall survival curves indicate that miR-27a-3p was differentially expressed between the normal and tumor groups, and had prognostic value (**Figures 7D, E**). Then, 136 emulative lncRNAs targeting miR-27a-3p were obtained from STARBASE; of these, AC107027.3 was the most significant (**Figure 7F**). In contrast to FASN, AC107027.3 expression was low in tumor tissues, and was positively correlated with overall survival (**Figures 7G, H**).

### Prediction of FASN-Targeting Drugs

Based on these molecular mechanism findings, we screened drugs targeting FASN and SREBF1 from the Cancer Therapeutics Response Portal (CTRP), Genomics of Drug Sensitivity in Cancer (GDSC), and CellMiner databases. In total, 23 and 61 drugs targeting FASN and SREBF1, respectively, were selected (correlation coefficient > 0 and *P* < 0.05; **Figure S11A**). Venn analysis between the different databases revealed that dasatinib and temsirolimus targeted FASN (**Figures 7I**, **S11B**), whereas navitoclax and PI-103 targeted SREBF1 (**Figure S11C**). Eight drugs targeted both FASN and SREBF1 (**Figure S10D**). Based on their IC_50_ scores, samples with different FASN expression responded differently to dasatinib and temsirolimus (**Figures 7J, K**). Based on information from canSAR Black, dasatinib and temsirolimus are FDA-approved drugs that are used mainly for tumor therapy (**Figures 7L, M**, **S10E, F**, **Table S3**).

## Discussion

**Figure 8:**
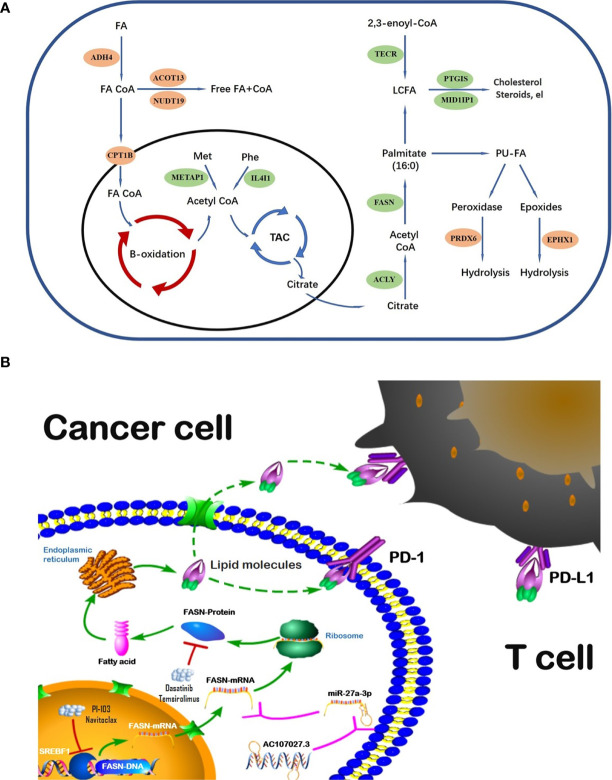
Schematic diagram of FAM genes **(A)** and FASN regulation **(B)**.

The global incident cases of bladder cancer have increased by more than 100 percent in the past few decades (32). Many gene signatures, including those for ferroptosis-related genes, autophagy-related genes, and hypoxia-related genes (33–35), have shown value in BC; nonetheless, we could find no prior studies using FAM-related models in BC prognosis and diagnosis. To address this gap, we evaluated the role of FAM genes in BC prognosis and immune infiltration. Our model identified 13 FAM genes (CPT1B, ACOT13, NUDT19, ADH4, EPHX1, and PRDX6, all catabolic; and FASN, MID1IP1, ACLY, PTGIS, METAP1, IL4I1, and TECR, all anabolic). The catabolic and anabolic FAM genes work together to regulate fatty acid metabolism and their FAM functions are shown in the schematic diagram (**Figure 8A**). Some of those genes have been reported to be involved in multiple cancers and others remain to be explored. For example, CPT1B, a protein for transporting pre-metabolites of fatty acids on the mitochondrial membrane, is a key target for controlling fatty acid beta-oxidation in mitochondria and has been reported as a therapeutic and grading target (18, 36). ACLY catalyzes the cleavage of citrate into the fatty acid synthesis substrate acetyl-CoA and is also a novel therapeutic target because of its function of glucose-to-acetate switch (37, 38). Because our model included the entire process of fatty acid synthesis and decomposition, it could effectively predict survival in BC.

We selected FASN, an important regulator in fatty acid anabolism, based on its prognostic signature. FASN was differentially expressed in tumor and normal tissues, was associated with tumor grade, and showed prognostic value in both the public and private databases that we studied. Its main function is to condense 7 malonyl-CoA molecules and 1 acetyl-CoA in series, to form the initial product of fatty acid synthesis, namely palmitate (15). FASN-dependent lipid metabolism influences neural stem cell proliferation and development (39). In recent years, increasing evidence has highlighted its important role in many cancers. High FASN expression is related to poor prognosis and metastasis in breast cancer (40, 41). In contrast, FASN inhibition both limits tumor–cell migration and invasiveness, and increases tumor sensitivity to drug therapy (42–44).This evidence, along with our findings, suggests that, while CPT1B is rate-limiting in fatty acid catabolism, FASN is the key regulator of fatty acid anabolism; together, they regulate the fatty acid metabolic pool and may play important role in progression and treatment of cancers.

Regulation analysis of FASN provides us more methods to modify fatty acid metabolism in BC (**Figure 8B**). Importantly, the TF most significantly involved in FASN regulation, SREBF1, also plays an important role in lipid metabolism (45, 46). Recent studies also reported that multiple lncRNAs competitively bind miRNAs to regulate FASN expression in nasopharyngeal and endometrial cancer (47, 48). For drugs targeting FASN, a phase II clinical trial revealed that temsirolimus show potential benefit in bladder cancer patients who are refractory to first line platinum-based chemotherapy (49). Our drug screening also revealed several drugs that target both SREBF1 and FASN, which may have better effects in BC patients.

Disrupted immunity in the TME plays an important role in cancer development and progression. Further, FAM is closely associated with immune-cell regulation in the TME (50, 51). In ovarian cancer, for instance, a FASN-related pathway was reported to disrupt dendritic cells and induce an impaired antitumor immune response *via* lipid accumulation (52). In our study, FASN was significantly associated with the immune microenvironment, immune-cell infiltration, and immune function in BC, and its CNV affected infiltration by CD4+ T cells, neutrophils, and dendritic cells. Most of the immune checkpoint genes (39/44) that we screened were differentially expressed between the high- and low-FASN groups. Further, ICI analysis revealed significant FASN expression in the CTLA4- and PD1-positive groups, with no significant effects in the double-negative groups. This suggests that, in BC patients, FASN expression is an indicator for anti-CTLA4 and anti-PD1 treatment. Prior studies (53–55) have indicated that many of the metabolic genes identified by our model, such as IL4I1, ACLY, and PTGIS, are involved in immune responses; however, while those studies considered gene expression and immune-cell infiltration, and its mechanisms, they did not consider immunotherapy. Our work addresses this gap.

## Conclusion

We constructed a prognostic risk-score model based on 13 FAM genes. The model effectively predicted prognosis in BC, independently of other clinicopathological features. It identified FASN as the key FAM gene in BC. FASN showed value in prognosis, and as an immunotherapy indicator and regulator, especially in anti-CTLA4 and anti-PD1 treatments. These findings present a novel way to predict prognosis in BC, and a novel target for ICI treatment. By describing the potential molecular mechanism whereby FASN functions, we provide support for further interventions using this target gene.

### Limitation

Although it revealed encouraging results, there are still several limitations in our study. First, the model was constructed and validated in a single data source (TCGA). It would be better if its prognostic value was tested in another independent patient cohort. Second, the original row data of the CH cohort used for validating the expression of the key gene was lost due to our lack of preservation awareness several years ago. However, we can provide all the data processing forms and previous studies based on these data. Last, the molecular mechanism used to regulate the key gene was not tested by more experiments, and we will continue to work on this in further studies.

## Data Availability Statement

The datasets presented in this study can be found in online repositories. The names of the repository/repositories and accession number(s) can be found in the article/**Supplementary Material**.

## Ethics Statement

The studies involving human participants were reviewed and approved by The Ethical Board of Changhai Hospital. The patients/participants provided their written informed consent to participate in this study. Written informed consent was obtained from the individual(s) for the publication of any potentially identifiable images or data included in this article.

## Author Contributions

QX proposed the project, conducted data analysis, interpreted the data, and wrote the manuscript. DF, ZW, YY, CX, and QW conducted data analysis, interpreted the data. SZ and LY supervised the project, and interpreted the data. All authors reviewed and edited the manuscript. All authors contributed to the article and approved the submitted version.

## Funding

This research was financed by grants from Qihang program of Naval Medical University, National Natural Science Foundation of China (81772720, 81802515, 81801854, 82172871, 81974099, 82170785, 81974098, 82170784).

## Conflict of Interest

The authors declare that the research was conducted in the absence of any commercial or financial relationships that could be construed as a potential conflict of interest.

## Publisher’s Note

All claims expressed in this article are solely those of the authors and do not necessarily represent those of their affiliated organizations, or those of the publisher, the editors and the reviewers. Any product that may be evaluated in this article, or claim that may be made by its manufacturer, is not guaranteed or endorsed by the publisher.

## References

[1] Sung H Ferlay J Siegel RL Laversanne M Soerjomataram I Jemal A. Global Cancer Statistics 2020: GLOBOCAN Estimates of Incidence and Mortality Worldwide for 36 Cancers in 185 Countries. CA Cancer J Clin (2021) 71:209–49. doi: 10.3322/caac.21660 33538338

[2] Zeng S Ying Y Xing N Wang B Qian Z Zhou Z. Noninvasive Detection of Urothelial Carcinoma by Cost-Effective Low-Coverage Whole-Genome Sequencing From Urine-Exfoliated Cell DNA. Clin Cancer Res (2020) 26:5646–54. doi: 10.1158/1078-0432.CCR-20-0401 33037018

[3] Ahmadi H Duddalwar V Daneshmand S. Diagnosis and Staging of Bladder Cancer. Hematol Oncol Clin North Am (2021) 35:531–41. doi: 10.1016/j.hoc.2021.02.004 33958149

[4] Montal ED Dewi R Bhalla K Ou L Hwang BJ Ropell AE. PEPCK Coordinates the Regulation of Central Carbon Metabolism to Promote Cancer Cell Growth. Mol Cell (2015) 60:571–83. doi: 10.1016/j.molcel.2015.09.025 PMC4656111 26481663

[5] Martínez-Reyes I Chandel NS. Cancer Metabolism: Looking Forward. Nat Rev Cancer (2021) 21:669–80. doi: 10.1038/s41568-021-00378-6 34272515

[6] Yoon H Shaw JL Haigis MC Greka A. Lipid Metabolism in Sickness and in Health: Emerging Regulators of Lipotoxicity. Mol Cell (2021) 81:3708–30. doi: 10.1016/j.molcel.2021.08.027 PMC8620413 34547235

[7] Broadfield LA Pane AA Talebi A Swinnen JV Fendt SM. Lipid Metabolism in Cancer: New Perspectives and Emerging Mechanisms. Dev Cell (2021) 56:1363–93. doi: 10.1016/j.devcel.2021.04.013 33945792

[8] Currie E Schulze A Zechner R Walther TC Farese RJ. Cellular Fatty Acid Metabolism and Cancer. Cell Metab (2013) 18:153–61. doi: 10.1016/j.cmet.2013.05.017 PMC3742569 23791484

[9] Tan Y Lin K Zhao Y Wu Q Chen D Wang J. Adipocytes Fuel Gastric Cancer Omental Metastasis *via* PITPNC1-Mediated Fatty Acid Metabolic Reprogramming. Theranostics (2018) 8:5452–68. doi: 10.7150/thno.28219 PMC6276097 30555557

[10] Sardesai SD Thomas A Gallagher C Lynce F Ottaviano YL Ballinger TJ. Inhibiting Fatty Acid Synthase With Omeprazole to Improve Efficacy of Neoadjuvant Chemotherapy in Patients With Operable TNBC. Clin Cancer Res (2021) 21:5810–7. doi: 10.1158/1078-0432.CCR-21-0493 34400413

[11] Chuang HY Lee YP Lin WC Lin YH Hwang JJ. Fatty Acid Inhibition Sensitizes Androgen-Dependent and -Independent Prostate Cancer to Radiotherapy *via* FASN/NF-κb Pathway. Sci Rep (2019) 9:13284. doi: 10.1038/s41598-019-49486-2 31527721 PMC6746859

[12] Zhang Y Kurupati R Liu L Zhou XY Zhang G Hudaihed A. Enhancing CD8(+) T Cell Fatty Acid Catabolism Within a Metabolically Challenging Tumor Microenvironment Increases the Efficacy of Melanoma Immunotherapy. Cancer Cell (2017) 32:377–91. doi: 10.1016/j.ccell.2017.08.004 PMC5751418 28898698

[13] Biswas S Lunec J Bartlett K. Non-Glucose Metabolism in Cancer Cells–Is It All in the Fat? Cancer Metastasis Rev (2012) 31:689–98. doi: 10.1007/s10555-012-9384-6 22706846

[14] Yu W Lei Q Yang L Qin G Liu S Wang D. Contradictory Roles of Lipid Metabolism in Immune Response Within the Tumor Microenvironment. J Hematol Oncol (2021) 14:187. doi: 10.1186/s13045-021-01200-4 34742349 PMC8572421

[15] Bogie J Haidar M Kooij G Hendriks J. Fatty Acid Metabolism in the Progression and Resolution of CNS Disorders. Adv Drug Deliv Rev (2020) 159:198–213. doi: 10.1016/j.addr.2020.01.004 31987838

[16] Gu Y Niu X Yin L Wang Y Yang Y Yang X. Enhancing Fatty Acid Catabolism of Macrophages Within Aberrant Breast Cancer Tumor Microenvironment Can Re-Establish Antitumor Function. Front Cell Dev Biol (2021) 9:665869. doi: 10.3389/fcell.2021.665869 33937269 PMC8081981

[17] Piyarathna D Rajendiran TM Putluri V Vantaku V Soni T von Rundstedt FC. Distinct Lipidomic Landscapes Associated With Clinical Stages of Urothelial Cancer of the Bladder. Eur Urol Focus (2018) 4:907–15. doi: 10.1016/j.euf.2017.04.005 PMC5650548 28753886

[18] Vantaku V Dong J Ambati CR Perera D Donepudi SR Amara CS. Multi-Omics Integration Analysis Robustly Predicts High-Grade Patient Survival and Identifies CPT1B Effect on Fatty Acid Metabolism in Bladder Cancer. Clin Cancer Res (2019) 25:3689–701. doi: 10.1158/1078-0432.CCR-18-1515 PMC6571061 30846479

[19] Zeng S Liu A Dai L Yu X Zhang Z Xiong Q. Prognostic Value of TOP2A in Bladder Urothelial Carcinoma and Potential Molecular Mechanisms. BMC Cancer (2019) 19:604. doi: 10.1186/s12885-019-5814-y 31216997 PMC6582551

[20] Lee JS Leem SH Lee SY Kim SC Park ES Kim SB. Expression Signature of E2F1 and Its Associated Genes Predict Superficial to Invasive Progression of Bladder Tumors. J Clin Oncol (2010) 28:2660–7. doi: 10.1200/JCO.2009.25.0977 20421545

[21] Dyrskjøt L Kruhøffer M Thykjaer T Marcussen N Jensen JL Møller K. Gene Expression in the Urinary Bladder: A Common Carcinoma *in Situ* Gene Expression Signature Exists Disregarding Histopathological Classification. Cancer Res (2004) 64:4040–8. doi: 10.1158/0008-5472.CAN-03-3620 15173019

[22] Hecker N Stephan C Mollenkopf HJ Jung K Preissner R Meyer HA. A New Algorithm for Integrated Analysis of miRNA-mRNA Interactions Based on Individual Classification Reveals Insights Into Bladder Cancer. PloS One (2013) 8:e64543. doi: 10.1371/journal.pone.0064543 23717626 PMC3663800

[23] Lindgren D Sjödahl G Lauss M Staaf J Chebil G Lövgren K. Integrated Genomic and Gene Expression Profiling Identifies Two Major Genomic Circuits in Urothelial Carcinoma. Plos One (2012) 7:e38863 22685613 10.1371/journal.pone.0038863 PMC3369837

[24] Sjödahl G Lauss M Lövgren K Chebil G Gudjonsson S Veerla S. A Molecular Taxonomy for Urothelial Carcinoma. Clin Cancer Res (2012) 18:3377–86. 10.1158/1078-0432.CCR-12-0077-T 22553347

[25] Hänzelmann S Castelo R Guinney J. GSVA: Gene Set Variation Analysis for Microarray and RNA-Seq Data. BMC Bioinf (2013) 14:7. doi: 10.1186/1471-2105-14-7 PMC3618321 23323831

[26] Subramanian A Tamayo P Mootha VK Mukherjee S Ebert BL Gillette MA. Gene Set Enrichment Analysis: A Knowledge-Based Approach for Interpreting Genome-Wide Expression Profiles. Proc Natl Acad Sci USA (2005) 102:15545–50. doi: 10.1073/pnas.0506580102 PMC1239896 16199517

[27] Lee BT Barber GP Benet-Pagès A Casper J Clawson H Diekhans M. The UCSC Genome Browser Database: 2022 Update. Nucleic Acids Res (2021) 50:1115–22. doi: 10.1093/nar/gkab959 PMC8728131 34718705

[28] Fornes O Castro-Mondragon JA Khan A van der Lee R Zhang X Richmond PA. JASPAR 2020: Update of the Open-Access Database of Transcription Factor Binding Profiles. Nucleic Acids Res (2020) 48:D87–92. doi: 10.1093/nar/gkz1001 PMC7145627 31701148

[29] Yang JH Li JH Shao P Zhou H Chen YQ Qu LH. Starbase: A Database for Exploring microRNA-mRNA Interaction Maps From Argonaute CLIP-Seq and Degradome-Seq Data. Nucleic Acids Res (2011) 39:D202–9. doi: 10.1093/nar/gkq1056 PMC3013664 21037263

[30] Liu CJ Hu FF Xia MX Han L Zhang Q Guo AY. GSCALite: A Web Server for Gene Set Cancer Analysis. Bioinformatics (2018) 34:3771–2. doi: 10.1093/bioinformatics/bty411 29790900

[31] Reinhold WC Sunshine M Liu H Varma S Kohn KW Morris J. CellMiner: A Web-Based Suite of Genomic and Pharmacologic Tools to Explore Transcript and Drug Patterns in the NCI-60 Cell Line Set. Cancer Res (2012) 72:3499–511. doi: 10.1158/0008-5472.CAN-12-1370 PMC3399763 22802077

[32] Zi H He SH Leng XY Xu XF Huang Q Weng H. Global, Regional, and National Burden of Kidney, Bladder, and Prostate Cancers and Their Attributable Risk Factors, 1990-2019. Mil Med Res (2021) 8:60. doi: 10.1186/s40779-021-00354-z 34819142 PMC8611255

[33] Sun J Yue W You J Wei X Huang Y Ling Z. Identification of a Novel Ferroptosis-Related Gene Prognostic Signature in Bladder Cancer. Front Oncol (2021) 11:730716. doi: 10.3389/fonc.2021.730716 34557413 PMC8455063

[34] Zhou C Li AH Liu S Sun H. Identification of an 11-Autophagy-Related-Gene Signature as Promising Prognostic Biomarker for Bladder Cancer Patients. Biology (Basel) (2021) 10:375. doi: 10.3390/biology10050375 33925460 PMC8146553

[35] Liu Z Tang Q Qi T Othmane B Yang Z Chen J. A Robust Hypoxia Risk Score Predicts the Clinical Outcomes and Tumor Microenvironment Immune Characters in Bladder Cancer. Front Immunol (2021) 12:725223. doi: 10.3389/fimmu.2021.725223 34484235 PMC8415032

[36] Abudurexiti M Zhu W Wang Y Wang J Xu W Huang Y. Targeting CPT1B as a Potential Therapeutic Strategy in Castration-Resistant and Enzalutamide-Resistant Prostate Cancer. PROSTATE (2020) 80:950–61. doi: 10.1002/pros.24027 32648618

[37] Lin R Tao R Gao X Li T Zhou X Guan KL. Acetylation Stabilizes ATP-Citrate Lyase to Promote Lipid Biosynthesis and Tumor Growth. Mol Cell (2013) 51:506–18. doi: 10.1016/j.molcel.2013.07.002 PMC4180208 23932781

[38] Zhao S Torres A Henry RA Trefely S Wallace M Lee JV. ATP-Citrate Lyase Controls a Glucose-To-Acetate Metabolic Switch. Cell Rep (2016) 17:1037–52. doi: 10.1016/j.celrep.2016.09.069 PMC5175409 27760311

[39] Knobloch M Braun SM Zurkirchen L von Schoultz C Zamboni N Araúzo-Bravo MJ. Metabolic Control of Adult Neural Stem Cell Activity by Fasn-Dependent Lipogenesis. NATURE (2013) 493:226–30. doi: 10.1038/nature11689 PMC3587167 23201681

[40] Breast Cancer Brain Metastases Rely on FASN-Mediated Lipid Biosynthesis. Cancer Discov (2021) 11:1315. doi: 10.1158/2159-8290.CD-RW2021-051 33837062

[41] Jiang W Xing XL Zhang C Yi L Xu W Ou J. MET and FASN as Prognostic Biomarkers of Triple Negative Breast Cancer: A Systematic Evidence Landscape of Clinical Study. Front Oncol (2021) 11:604801. doi: 10.3389/fonc.2021.604801 34123778 PMC8190390

[42] Gruslova A McClellan B Balinda HU Viswanadhapalli S Alers V Sareddy GR. FASN Inhibition as a Potential Treatment for Endocrine-Resistant Breast Cancer. Breast Cancer Res Treat (2021) 187:375–86. doi: 10.1007/s10549-021-06231-6 33893909

[43] Humbert M Seiler K Mosimann S Rentsch V Sharma K Pandey AV. Reducing FASN Expression Sensitizes Acute Myeloid Leukemia Cells to Differentiation Therapy. Cell Death Differ (2021) 28:2465–81. doi: 10.1038/s41418-021-00768-1 PMC8329134 33742137

[44] Papaevangelou E Almeida GS Box C DeSouza NM Chung YL. The Effect of FASN Inhibition on the Growth and Metabolism of a Cisplatin-Resistant Ovarian Carcinoma Model. Int J Cancer (2018) 143:992–1002. doi: 10.1002/ijc.31392 29569717 PMC6055739

[45] Li LY Yang Q Jiang YY Yang W Jiang Y Li X. Interplay and Cooperation Between SREBF1 and Master Transcription Factors Regulate Lipid Metabolism and Tumor-Promoting Pathways in Squamous Cancer. Nat Commun (2021) 12:4362. doi: 10.1038/s41467-021-24656-x 34272396 PMC8285542

[46] Wang X Sato R Brown MS Hua X Goldstein JL. SREBP-1, A Membrane-Bound Transcription Factor Released by Sterol-Regulated Proteolysis. Cell (1994) 77:53–62. doi: 10.1016/0092-8674(94)90234-8 8156598

[47] Liu F Wei J Hao Y Lan J Li W Weng J. Long Intergenic Non-Protein Coding RNA 02570 Promotes Nasopharyngeal Carcinoma Progression by Adsorbing microRNA miR-4649-3p Thereby Upregulating Both Sterol Regulatory Element Binding Protein 1, and Fatty Acid Synthase. Bioengineered (2021) 12:7119–30. doi: 10.1080/21655979.2021.1979317 PMC8806647 34546840

[48] He Y Xu S Qi Y Tian J Xu F. Long Noncoding RNA SNHG25 Promotes the Malignancy of Endometrial Cancer by Sponging microRNA-497-5p and Increasing FASN Expression. J Ovarian Res (2021) 14:163. doi: 10.1186/s13048-021-00906-w 34789312 PMC8600866

[49] Pulido M Roubaud G Cazeau AL Mahammedi H Vedrine L Joly F. Safety and Efficacy of Temsirolimus as Second Line Treatment for Patients With Recurrent Bladder Cancer. BMC Cancer (2018) 18:194. doi: 10.1186/s12885-018-4059-5 29454321 PMC5816357

[50] Lochner M Berod L Sparwasser T. Fatty Acid Metabolism in the Regulation of T Cell Function. Trends Immunol (2015) 36:81–91. doi: 10.1016/j.it.2014.12.005 25592731

[51] Wu H Han Y Rodriguez SY Deng H Siddiqui S Treese C. Lipid Droplet-Dependent Fatty Acid Metabolism Controls the Immune Suppressive Phenotype of Tumor-Associated Macrophages. EMBO Mol Med (2019) 11:e10698. doi: 10.15252/emmm.201910698 31602788 PMC6835560

[52] Jiang L Fang X Wang H Li D Wang X. Ovarian Cancer-Intrinsic Fatty Acid Synthase Prevents Anti-Tumor Immunity by Disrupting Tumor-Infiltrating Dendritic Cells. Front Immunol (2018) 9:2927. doi: 10.3389/fimmu.2018.02927 30619288 PMC6302125

[53] Romagnani S. IL4I1: Key Immunoregulator at a Crossroads of Divergent T-Cell Functions. Eur J Immunol (2016) 46:2302–5. doi: 10.1002/eji.201646617 27726138

[54] Xu Y Zhang Z Xu D Yang X Zhou L Zhu Y. Identification and Integrative Analysis of ACLY and Related Gene Panels Associated With Immune Microenvironment Reveal Prognostic Significance in Hepatocellular Carcinoma. Cancer Cell Int (2021) 21:409. doi: 10.1186/s12935-021-02108-2 34344378 PMC8335999

[55] Dai D Chen B Feng Y Wang W Jiang Y Huang H. Prognostic Value of Prostaglandin I2 Synthase and Its Correlation With Tumor-Infiltrating Immune Cells in Lung Cancer, Ovarian Cancer, and Gastric Cancer. Aging (Albany NY) (2020) 12:9658–85. doi: 10.18632/aging.103235 PMC7288932 32463792

