# Supplementary material for: Fatty Acid Synthase Is the Key Regulator of Fatty Acid Metabolism and Is Related to Immunotherapy in Bladder Cancer
Source: Front Immunol. 2022 Mar 22;13:836939. doi: 10.3389/fimmu.2022.836939 (PMC8982515; doi:10.3389/fimmu.2022.836939)
Supplement: Supplementary file 1 [file DataSheet_1.docx]

**Supplementary materials**

**1.Construction and validation of risk model.**

## Tumor modification based on prognostic FAMGs using consensus clustering

Consensus clustering was employed to explore the connection of prognostic FAMGs expression and BC subgroups using “ConsensusClusterPlus” package. We found that when clustering variable k=2, the intragroup correlations were the highest and the intergroup correlations were the lowest (Fig.S2A-2C). Meanwhile, CDF curve, delta area and tracking plot also revealed that clustering variable k=2 was the best parameter which means that the samples would be better to be divided into 2 subtypes (Fig.S2D-2F). PCA analysis showed that the samples were obviously distinguished between the different clusters (Fig.S2G). Differentially expressed analysis between the two subgroups found 1077 DEGs for further study (Fig.S2H). Relationship of prognostic gene expression and the clinicopathology features involved age, gender, stage, grade, and TNM stage was analyzed and presented on a heatmap (Fig.S2I), demonstrating that gene expression and clinical features were also differentially enriched in cluster 1 and 2. Kaplan-Meier OS curve of the two subgroups indicated that cluster 1 had a better prognosis compared to cluster 2 (Fig.S2J).

## Construction and Validation of fatty acid metabolism-related model

To construct a more efficient fatty acid metabolism-related model, we used LASSO regression analysis repeatedly and screened out interfering genes manually in training cohort. Based on the 68 prognostic FAMGs, 24 and 15 genes were identified in the first and second LASSO cycles, respectively (Fig.S3A-S3D). Another two genes were then excluded by the following multivariate regression analysis and finally identified 13 genes (CPT1B, FASN, MID1IP1, ACOT13, ACLY, NUDT19, TECR, PTGIS, ADH4, PRDX6, IL4I1, EPHX1, METAP1) to construct the risk model (Fig.S3E). The correlation analysis of the 13 candidate genes were also demonstrated (Fig.S3F).

The risk score was calculated by the following formula：Risk score = 0.2392*FASN - 0.3985*CPT1B - 0.2063*MID1IP1 - 0.5235*ACOT13 + 0.3153*ACLY - 0.3382*NUDT19 + 0.3508*TECR + 0.0705*PTGIS - 0.2288*ADH4 + 0.2959*PRDX6 - 0.2468*IL4I1 + 0.2116*EPHX1 - 0.2395*METAP1. All samples were divided into high and low risk groups by risk scores. Further analyses shown that the survival status was significant different between high and low risk groups (Fig. S3G, S3H).

The model constructed in training cohort was then validated in both training, testing and entire cohort. The AUC of 1-, 3-, and 5-year OS predicted with the model in training cohort were 0.802, 0.744 and 0.803 respectively (Fig. S3I). Kaplan-Meier analysis indicated that there was a significant difference between high and low risk groups (Fig.S3J). Risk plot shown that patients with higher risk score were associated with higher mortality and shorter survival time (Fig.S3K). In addition, differential expression analysis revealed that 7 genes (CPT1B, MID1IP1, ACOT13, NUDT19, ADH4, IL4I1) mainly enriched in low-risk group and other 6 genes mainly enriched in high-risk group (Fig.S3L). In testing cohort, The AUC of 1-, 3-, and 5-year OS were 0.745, 0.771 and 0.780 respectively (Fig.S3M). OS, survival status and gene expression were also different between high and low risk groups (Fig. S3N-S3P). Similar results were also obtained in entire cohort (Fig.S3Q-S3T).

## Nomogram

Univariate and multivariate cox analysis in TCGA cohort both revealed that risk score was an independent predictor for BC patients (Fig.S4A, S4B). A heatmap describing the relationship of clinicopathological features (age, gender, stage, grade, cluster, and TNM stage) and risk group, which indicated that T, N, M, stage and cluster was significant different between high and low risk group (Fig.S4C). The nomogram based on risk group and clinicopathological features was bult to predict survival status and 1-, 3- and 5-year survival rates of BC patients were 86.2%, 59.3% and 52.4% (Fig.S4D). The calibration curves confirmed that the nomogram was relatively reliable in predicting the prognosis of BC patients (Fig.S4E). In addition, the risk core was proved to be more efficient than other clinicopathological features and cluster in predicting the AUC (Fig.S4F) and decision curve analysis (DCA) analysis showed that combining the risk score with clinicopathological features could increase the net benefit of evaluating the outcome of BC patients (Fig.S4G).

## CNV, TBM and immune infiltration of the model

CNV status of the 13 model genes was analyzed by R and Strawberry software. The location and CNV frequency of the 13 model genes were presented on a circle diagram of chromosome (Fig.S5A). ACOT13 had the highest frequency of copy number amplification and CPT1B had the highest frequency of copy number deletion (Fig. S5B). TBM analysis found that 13 model genes altered in 8.09% (33/408) TCGA-BLCA patients and FASN had the highest mutation frequency (2%) (Fig.S5C) but no difference of TBM was discovered between high and low risk group (Fig.S5D).

Analyzed by seven algorithms (TIMER, CIBERSORT, CIBERSORT-ABS, QUANTISEQ, MCPCOUNTER, XCELL, and EPIC), differential expression of immune cells between two risk groups was presented as a heatmap (Fig.S5E). Violin plot of immune cells infiltration in CIBERSORT shown that B cells naïve, M0, M2 and Mast cells resting were enriched in high-risk group whereas T cells CD8, T cells CD4 memory activated, T cells follicular helper, T cells regulatory and Dendritic cells activated were enriched in low-risk group (Fig. S5F). Evaluation of ICI therapy based on TCIA revealed that patients in low-risk group had better response to immune checkpoint inhibitors treatments than high-risk group (Fig.S5G).

**2.Supplementary pictures.**

## Figure S1


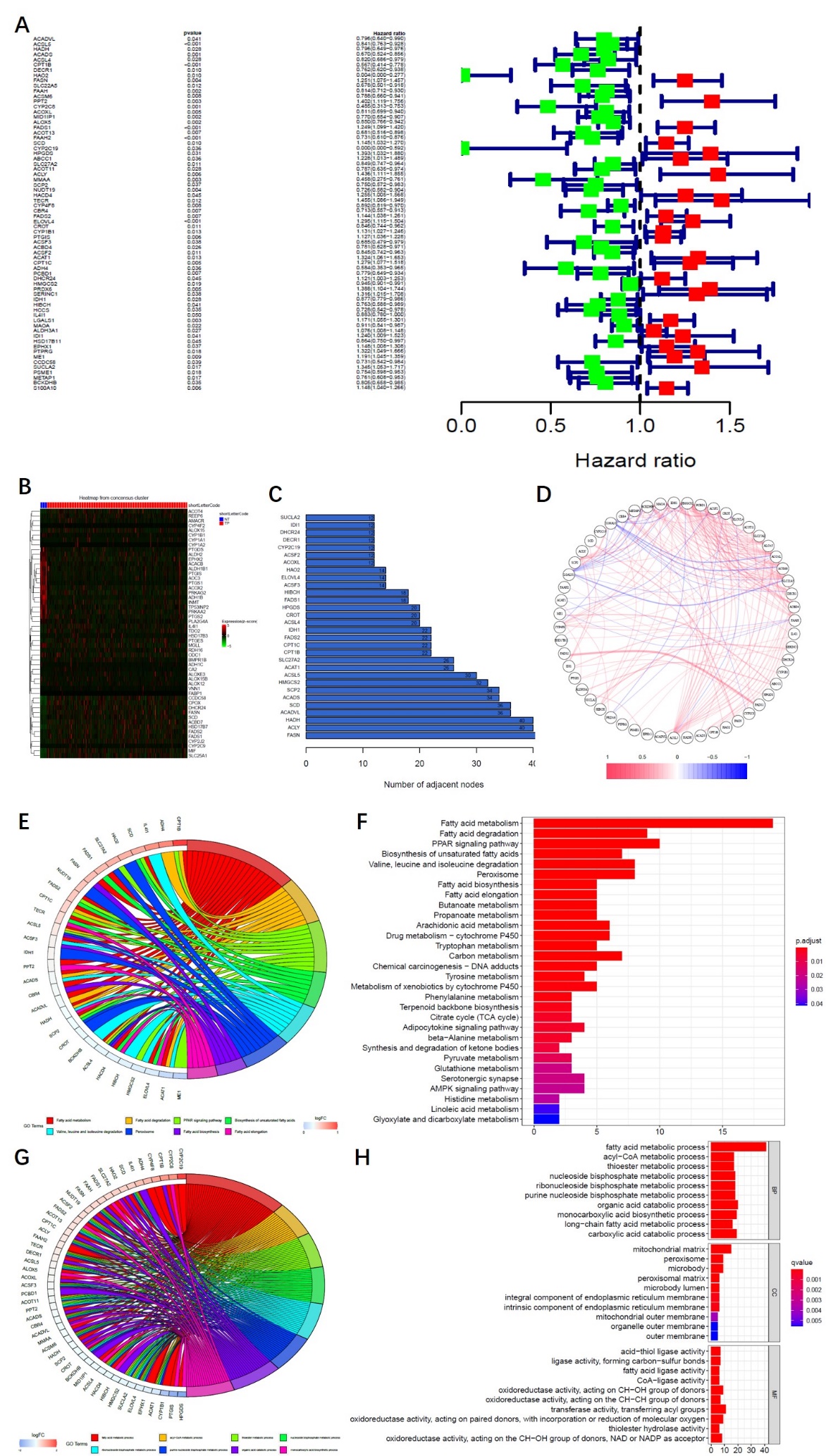


**Identification of differentially expressed and prognostic FAMGs. (A)** Univariate Cox regression identified 68 prognostic FAMGs (P < 0.05). (**B)** Volcano plot of differentially expressed FAMGs (logFC<1, p<0.05). (**C)** Adjacent nodes of the hub genes in PPI network. (**D)** The correlation network of the 68 prognostic FAMGs (red line: positive correlation; blue line: negative correlation. P<0.05). (**E-F)** KEGG pathways of the 68 prognostic FAMGs. (**G-H)** GO function enrichment of the 68 prognostic FAMGs.

## Figure S2


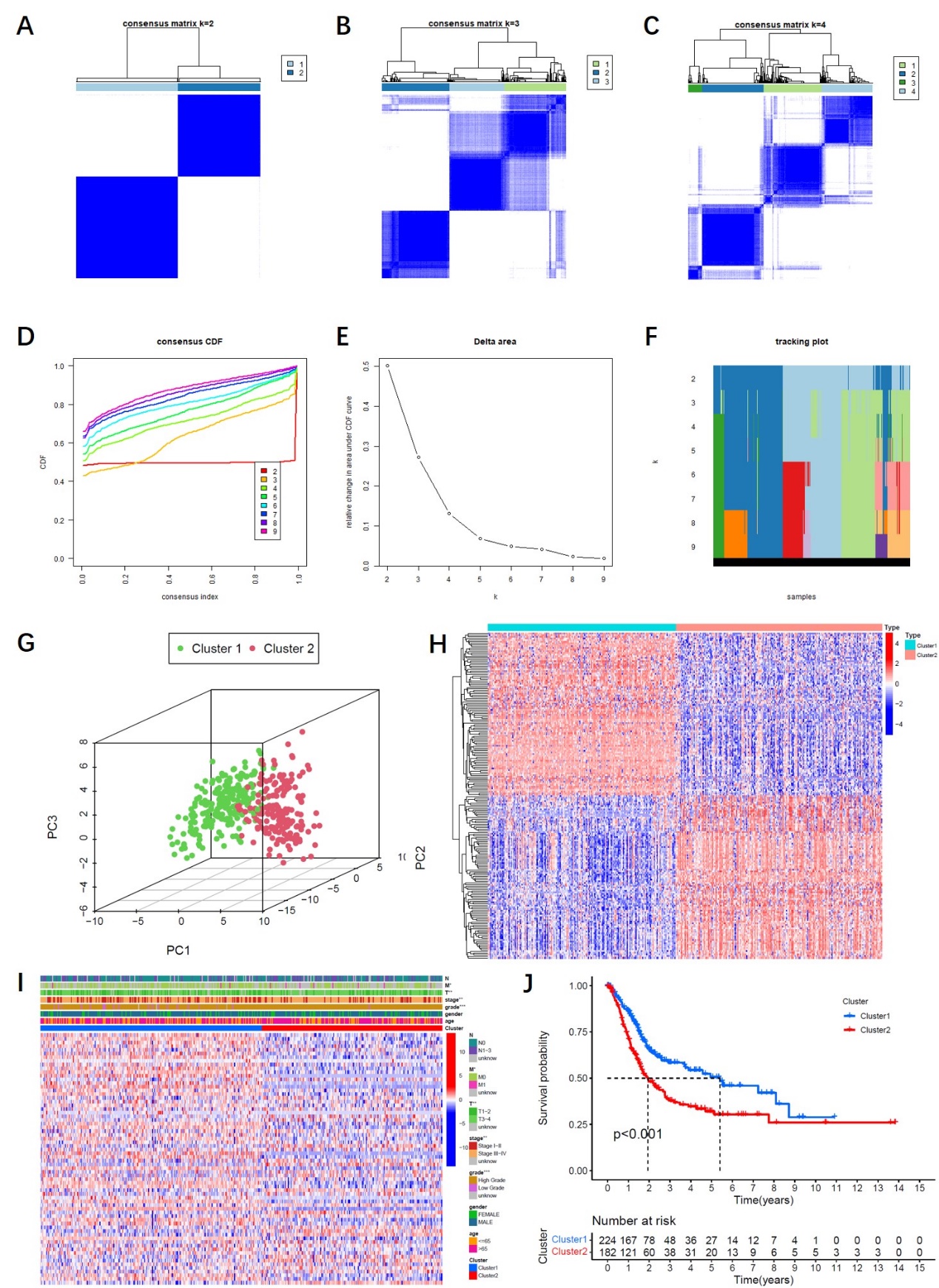


**Subgroup analysis based on prognostic FAMGs. (A-C)** Consensus matrix of different clustering variable (k=2, 3, and 4). **(D-F)** CDF curve, delta area and tracking plot of different clustering variable (k=2-9). **(G)** Three dimensional PCA diagram of the 2 subgroups (194 samples in cluster 1 and 212 samples in cluster 2). **(H)** Heatmap of differentially expressed genes between two subgroups. **(I)** Heatmap of prognostic FAMGs between two subgroups and clinicopathology features. **(J)** Overall survival analysis of the two subgroups based on 406 patients from TCGA.

## Figure S3


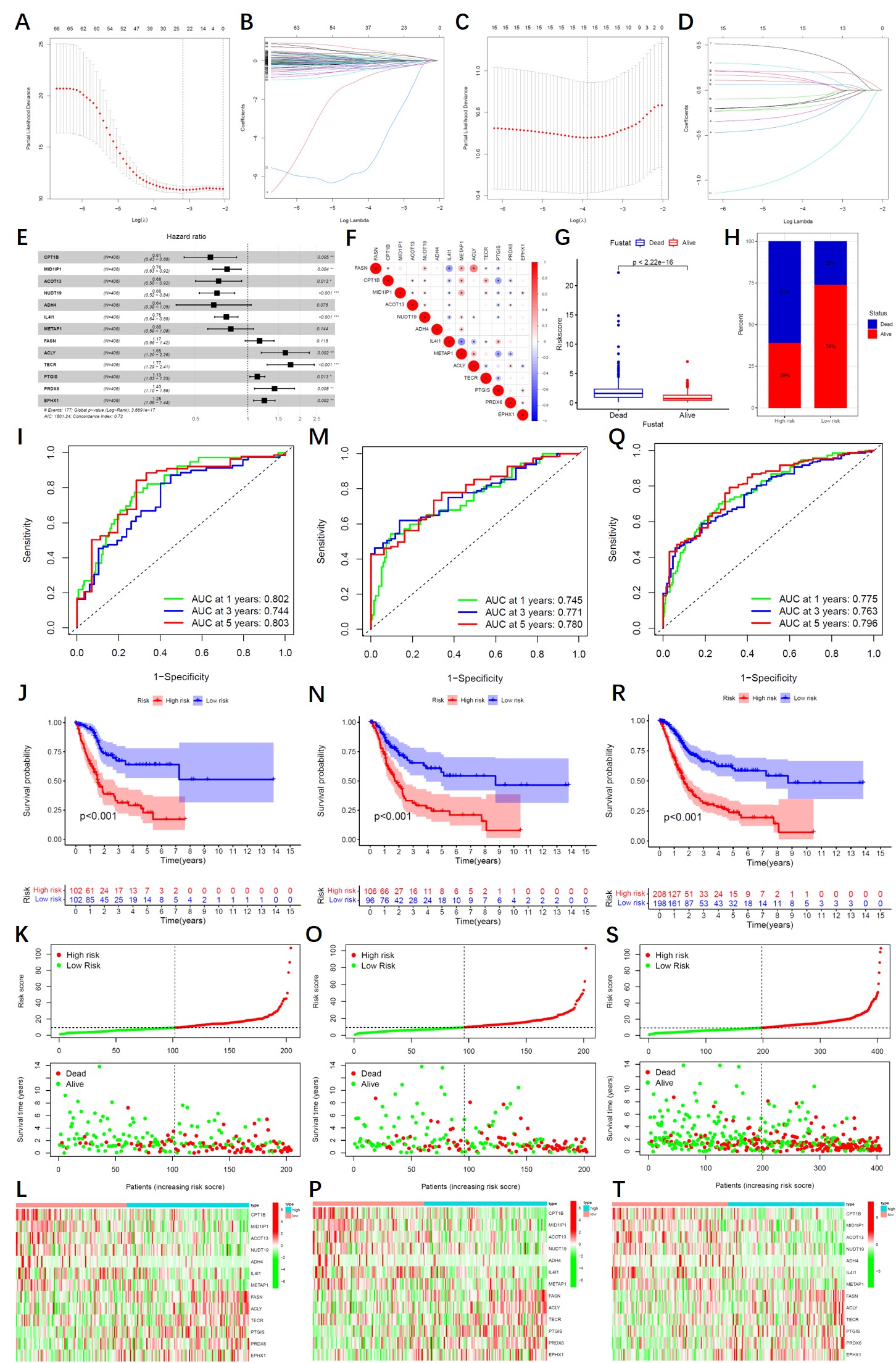


**Construction and validation of fatty acid metabolism-related model. (A-D)** Dynamic coefficient profiling and Partial likelihood deviation in the first and second LASSO analysis (the dashed lines on the left and right indicated the “lambda. min” and “lambda.1se” criteria). **(E)** Forest plot of the candidate genes by multivariate analysis. **(F)** Correlation diagram of candidate genes (p<0.05). **(G, H)** Association of survival status and risk scores and groups. **(I-L)** Validation of risk model in training cohort. **(M-P)** Validation of risk model in testing cohort. **(Q-T)** Validation of risk model in entire cohort.

## Figure S4


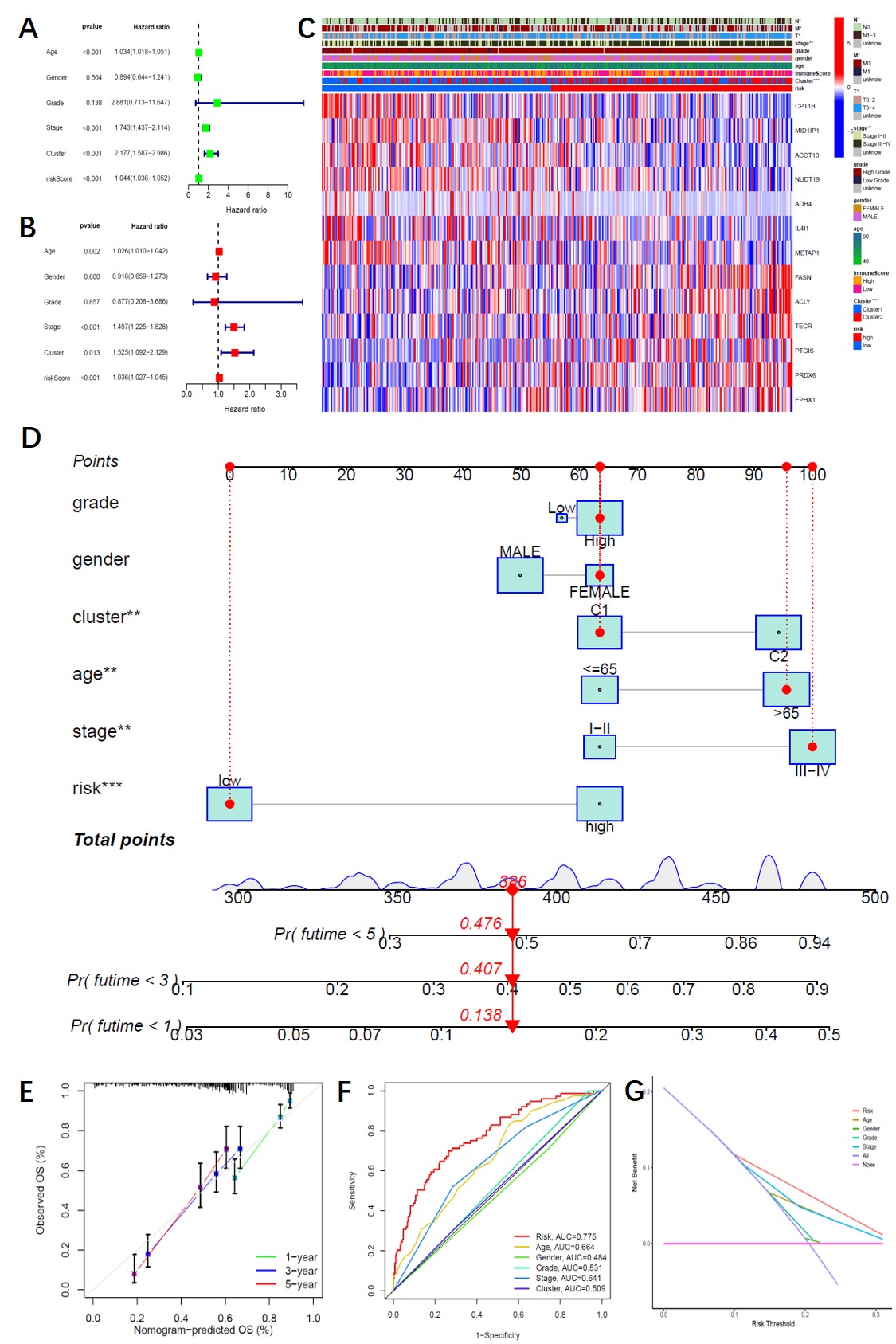


**Nomogram of risk score and clinicopathology features. (A)** Univariate analysis of risk score in TCGA. **(B)** Multivariate analysis of risk score in TCGA. **(C)** Heatmap of connections between risk group and clinicopathologic features (*p<0.05). **(D)** Nomogram of risk groups and clinicopathologic features in TCGA (*p<0.05). **(E)** Calibration curves of the nomogram in 1-, 3-, and 5- years. **(F)** Relative AUC of risk group and clinicopathologic features. **(G)** DCA analysis of risk group and clinicopathologic features.

## Figure S5


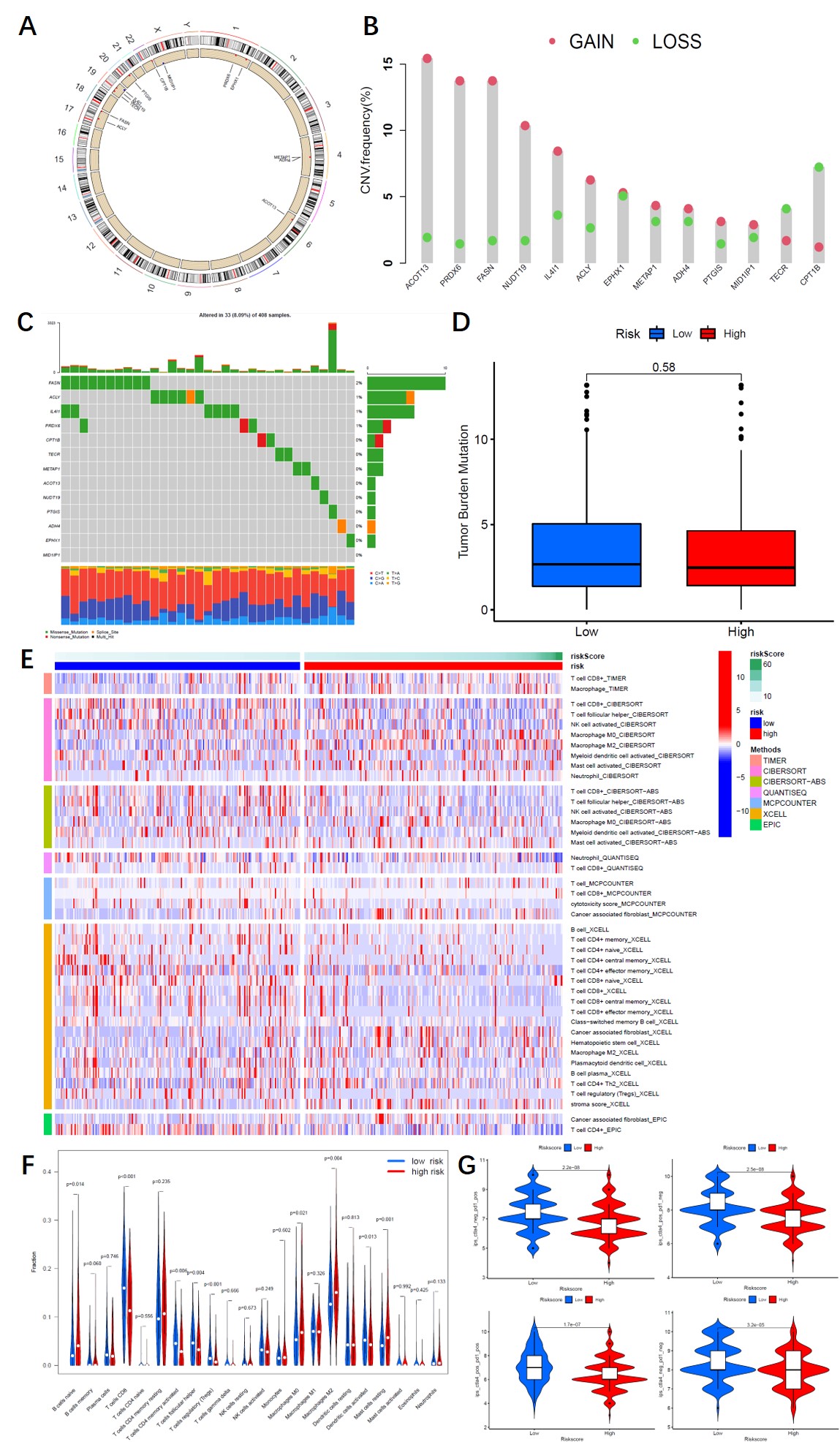


**Function analysis of the risk score model. (A)** CNV status of the candidate genes on chromosomes. **(B)** CNV frequency of gain and loss in 13 candidate genes. **(C)** Mutation frequency of the 13 candidate genes in 408 TCGA patients. **(D)** Boxplot of TBM analysis between low and high-risk groups. **(E)** Heatmap of immune infiltration between low and high-risk groups by seven algorithms (p<0.05). **(F)** Differential expression of immune cells infiltration between low and high-risk groups. **(G)** Different response of ICI therapy between low and high-risk groups (anti-CTLA4 and anti-PD1).

## Figure S6


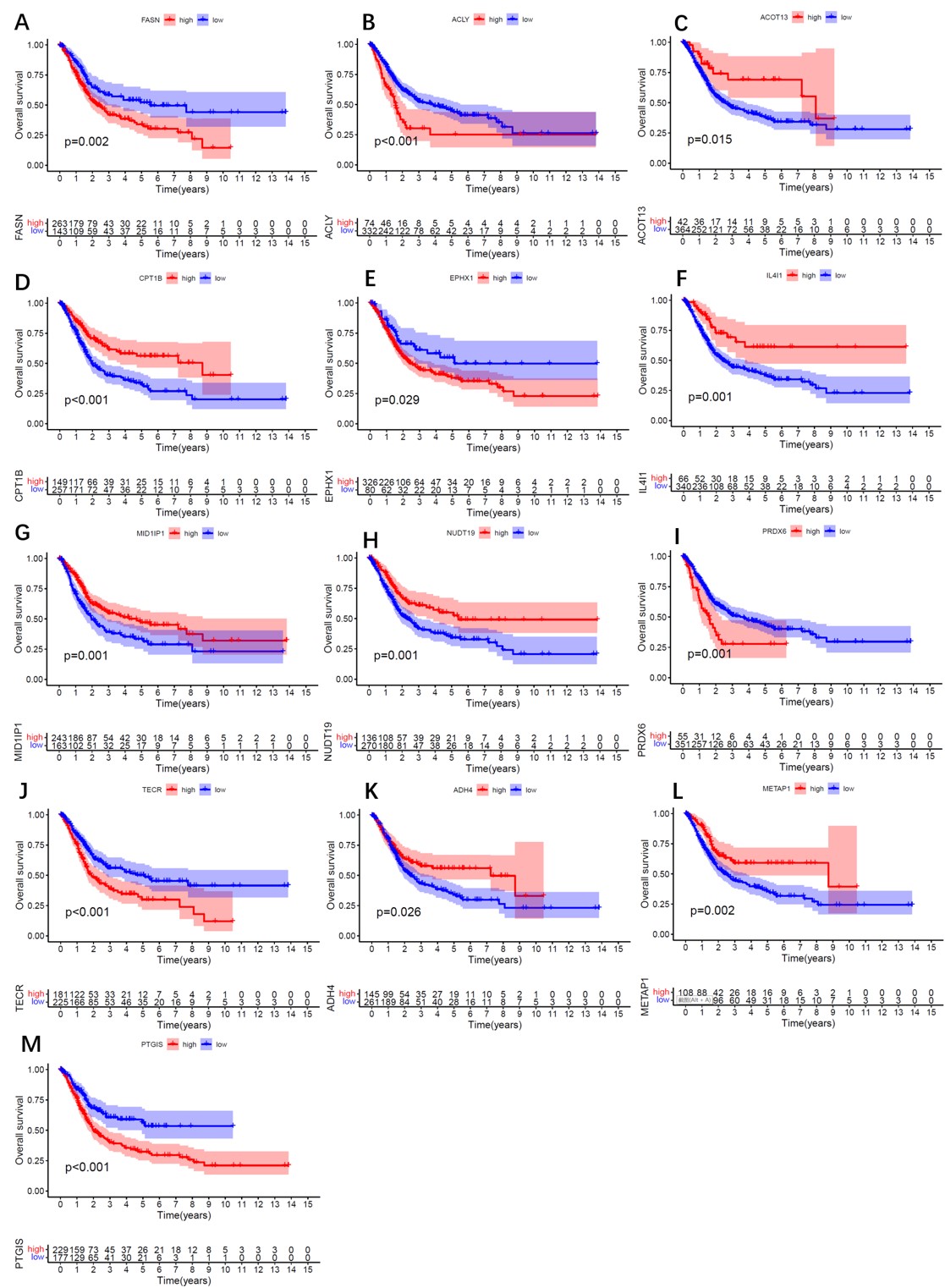


KM analysis of the 13 candidate genes between low and high expression groups in TCGA**.**

## Figure S7


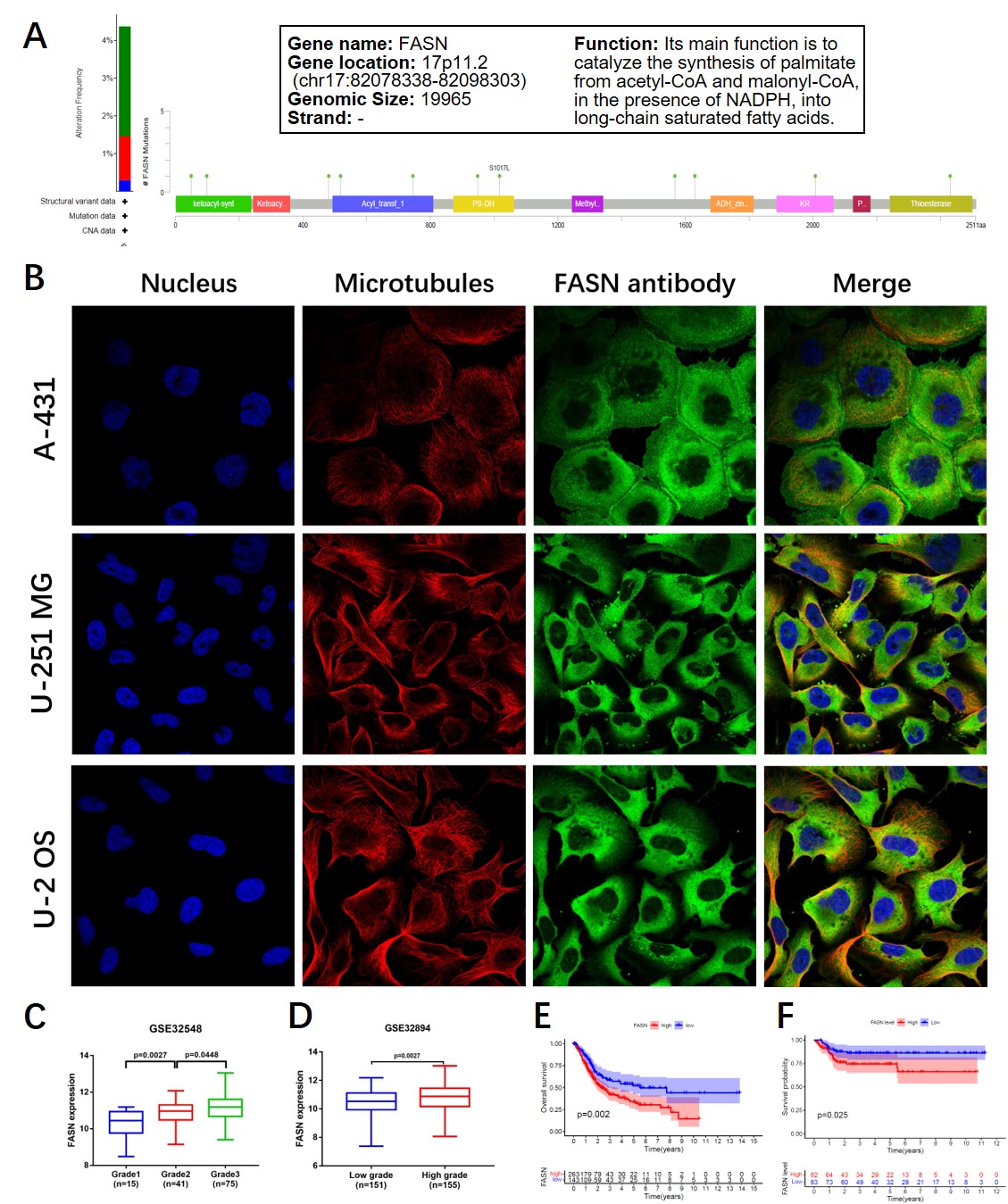


**Validation of FASN. (A)** Basic information and mutation data of FASN. **(B)** Immunofluorescence images of FASN in A-431 (Epidermoid carcinoma), U-251 MG (Glioblastoma) and U-2 OS (Osteosarcoma). **(C, D)** Differential expression of FASN between different grade patients in GSE32548 and GSE32894. (**E**) Overall survival curve of BC patients in TCGA grouped by the optimal cutoff value. (F) Disease-free survival curve of BC patients in GSE13507.

## Figure S8


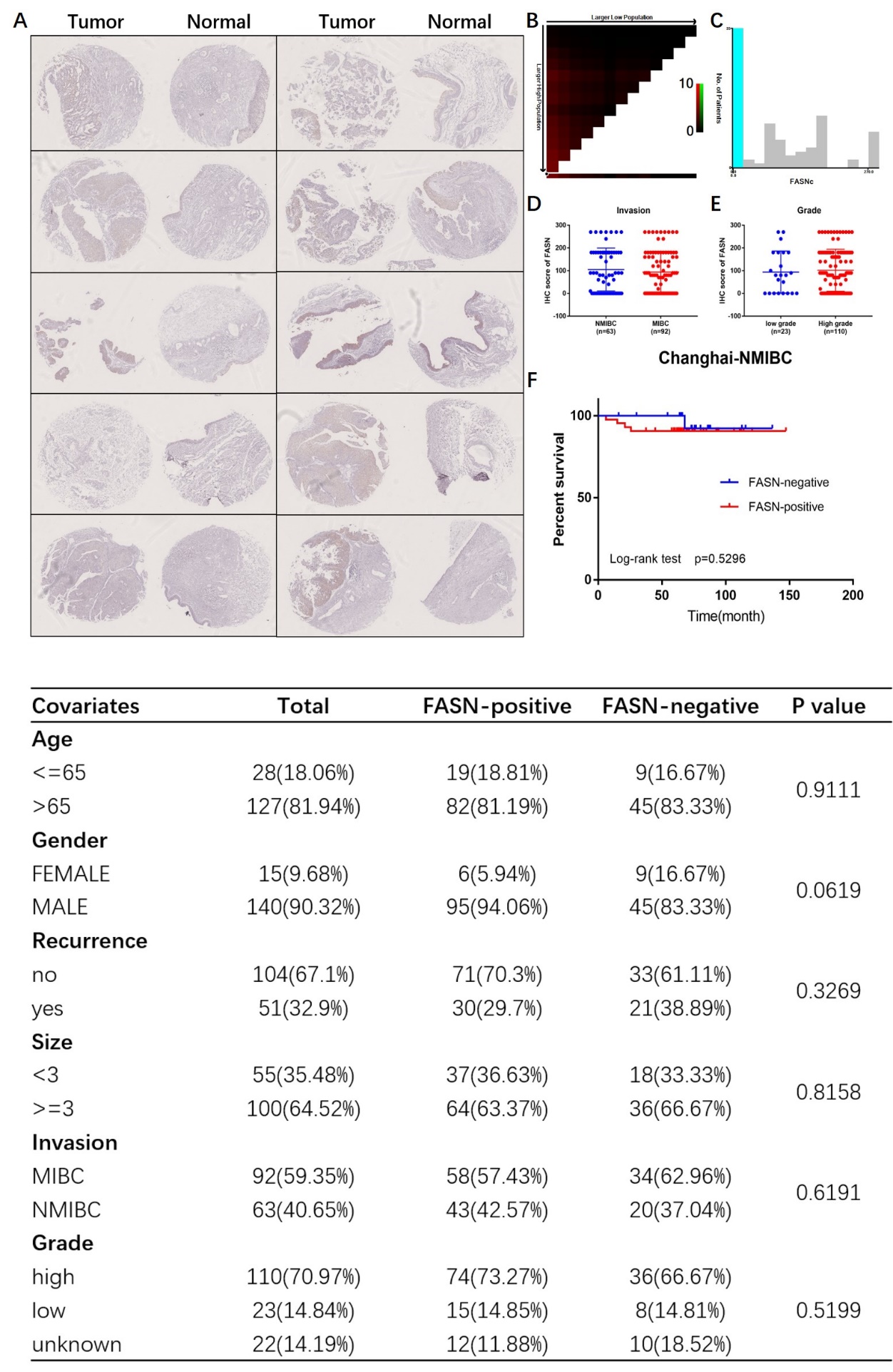


**External validation of FASN. (A)** IHC staining of the 10 paired normal and tumor tissues in CH cohort. **(B, C)** IHC scores of FASN and patients grouping. **(D, E)** Differential analysis of FASN in different invasion and grade groups. **(F)** KM overall survival curve between positive and negative-FASN groups in NMIBC patients of CH cohort.

## Figure S9

**
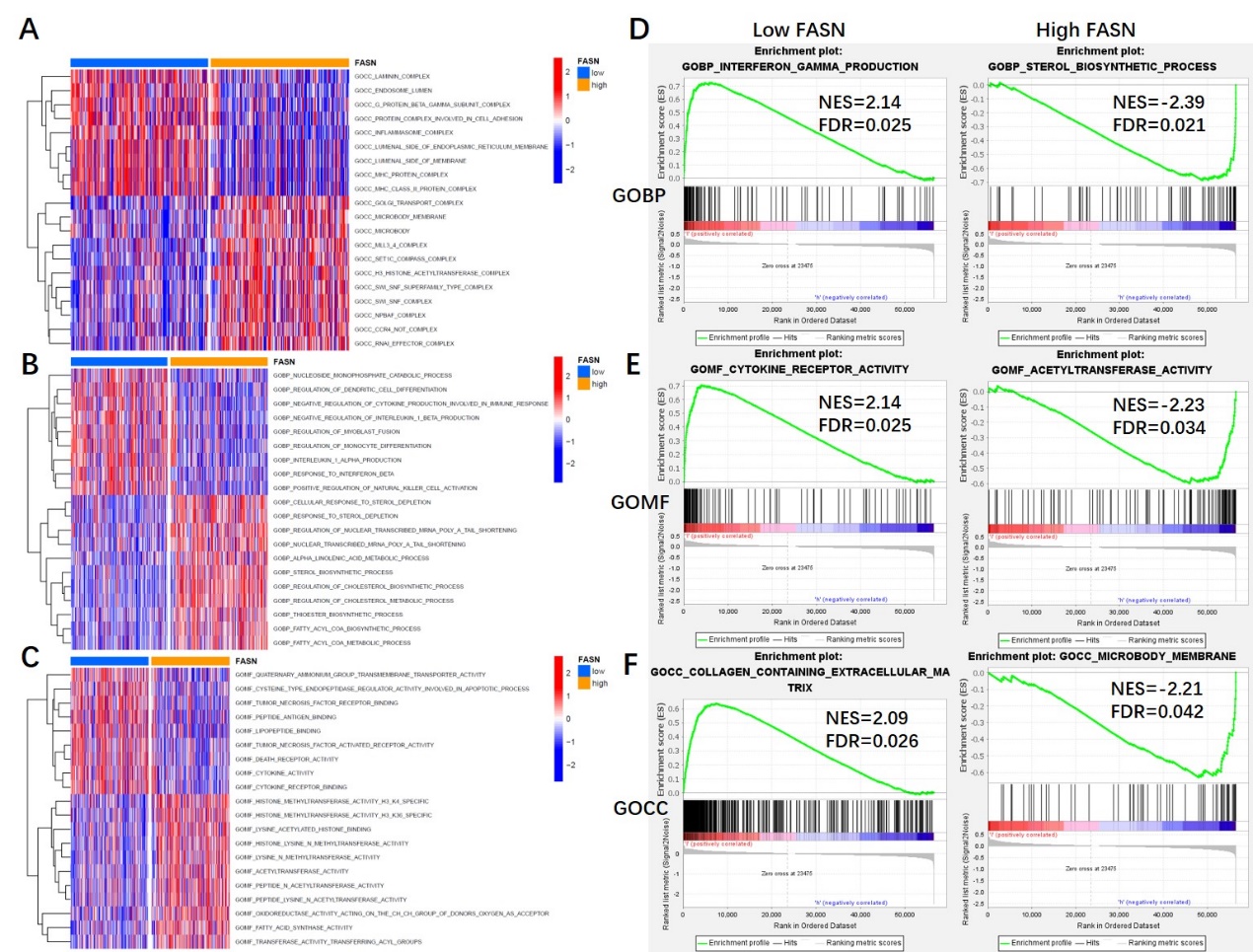
**

**GSVA and GSEA. (A-C)** GOCC, GOBP and GOMF function enrichment in low and high-FASN groups in GSVA (p<0.05). **(D-F)** Significant top pathway of GOCC, GOBP and GOMF enriched in low and high-FASN groups in GSEA (p<0.05).

## Figure S10

**
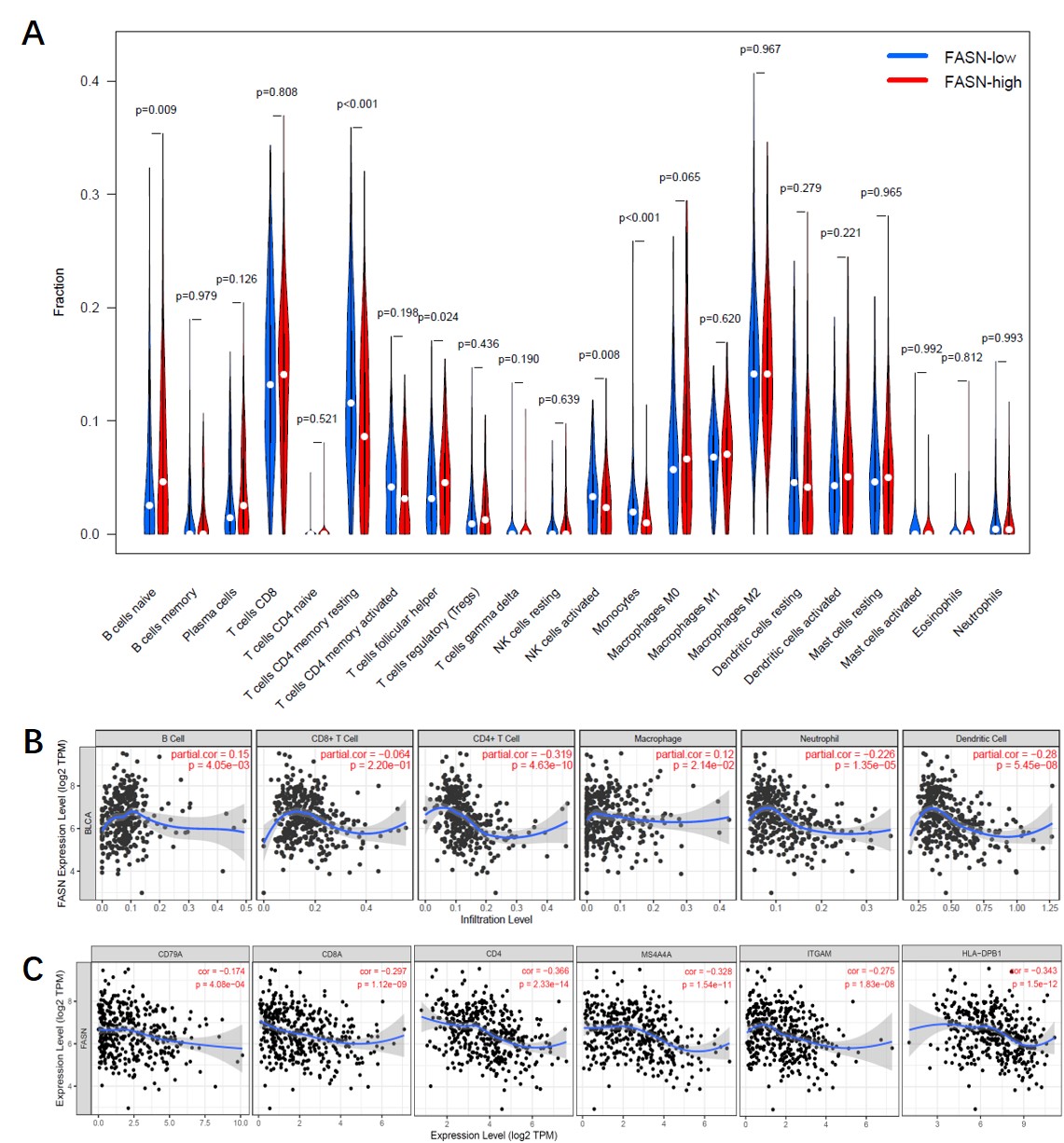
**

**Immune function of FASN. (A)** Differential expression of immune cells infiltration between low- and high-FASN groups. **(B)** Correlation analysis of FASN and immune cells infiltration in BC. **(C)** Correlation analysis of FASN and marker gene of immune cells.

## Figure S11


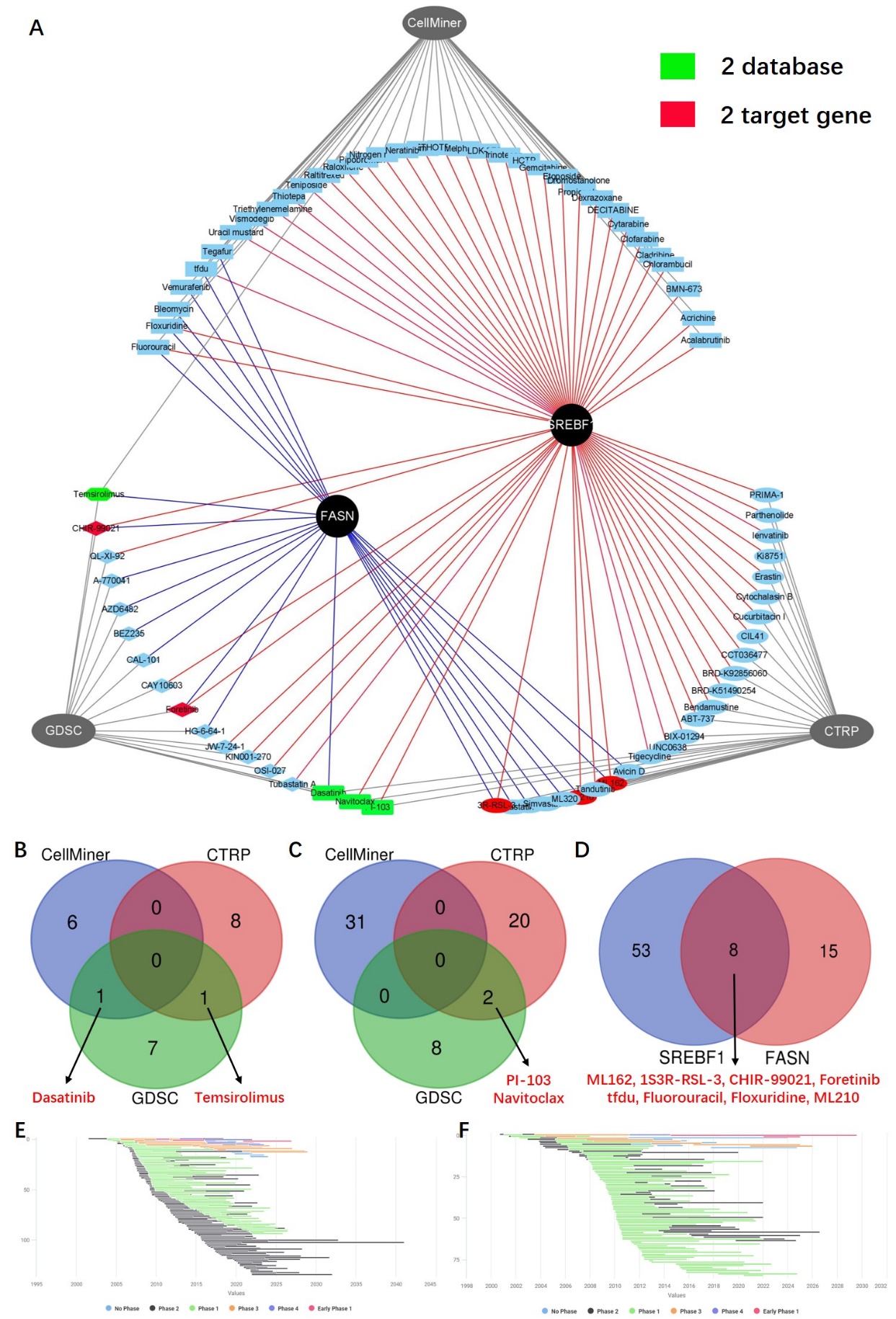


**Drug network of FASN and SREBF1. (A)** Network of drugs respectively targeting FASN and SREBF1 in CTRP, GDSC and CellMiner ([coefficient](javascript:;) > 0, p value < 0.05). **(B)** Venn plot of drugs targeting FASN in CTRP, GDSC and CellMiner. **(C)** Venn plot of drugs targeting SREBF1 in CTRP, GDSC and CellMiner. **(D)** Venn plot of drugs targeting both FASN and SREBF1. **(E, F)** Information of clinical studies based on Dasatinib and Temsirolimus from canSARblack.

**3.Supplementary tables.**

**Table S1-13 model genes**

**Table S2-clinicoFASN of CH**

**Table S3-clinical study of Dasatinb and Temsirolimus**

**Table S4-ICI score**
